# Evaluation of the Safety and Regenerative Potential of Human Mesenchymal Stem Cells and Their Extracellular Vesicles in a Transgenic Pig Model of Cartilage-Bone Injury In Vivo – Preclinical Study

**DOI:** 10.1007/s12015-025-10853-4

**Published:** 2025-05-17

**Authors:** Anna Łabędź-Masłowska, Jarosław Wieczorek, Maciej Mierzwiński, Małgorzata Sekuła-Stryjewska, Sylwia Noga, Jolanta Rajca, Piotr Duda, Katarzyna Milian-Ciesielska, Elżbieta Karnas, Katarzyna Kmiotek-Caller, Agnieszka Szkaradek, Zbigniew Madeja, Krzysztof Ficek, Jacek Jura, Ewa Zuba-Surma

**Affiliations:** 1 Department of Cell Biology, Faculty of Biochemistry, Biophysics and Biotechnology, Jagiellonian University, Krakow, Poland; 2 University Center of Veterinary Medicine UJ-UR, University of Agriculture in Krakow, Krakow, Poland; 3 Department of Science, Innovation and Development, Galen-Orthopaedics, Bierun, Poland; 4 Laboratory of Stem Cell Biotechnology, Malopolska Centre of Biotechnology, Jagiellonian University, Krakow, Poland; 5 Spin-Lab Centre for Microscopic Research on Matter, University of Silesia in Katowice, Katowice, Poland; 6 Institute of Biomedical Engineering, Faculty of Science and Technology, University of Silesia in Katowice, Katowice, Poland; 7 Department of Pathomorphology, Jagiellonian University Medical College, University Hospital, Krakow, Poland; 8 Department of Reproductive Biotechnology and Cryoconservation, National Research Institute of Animal Production, Balice, Poland

**Keywords:** Adipose tissue-derived mesenchymal stem/stromal cells, Umbilical cord-derived mesenchymal stem/stromal cells, Extracellular vesicles, Osteoarthritis, Transgenic pig model of cartilage-bone injury, ATMPs

## Abstract

Osteoarthritis (OA) is a degenerative joint condition leading to disability. The lack of effective treatment for OA creates a need for the development of new therapeutic approaches that may rely on stem cells including mesenchymal stem/stromal cells (MSCs) and their derivatives such as extracellular vesicles (EVs). The objective of this study was to evaluate the impact of MSCs derived from adipose tissue (AT-MSCs) and umbilical cord (UC-MSCs) and their EVs on cartilage-bone injury in vivo, to identify the specimen with the highest regenerative potential for further clinical applications in patients with OA. Humanized pigs underwent cartilage-bone injuries followed by intraarticular administration of products containing AT-MSCs, UC-MSCs, AT-MSC-EVs or UC-MSC-EVs mixed with hyaluronic acid (HA) or HA alone (for comparison). After 6-m follow-up, almost-fully-healed cartilage-bone defects were observed in the AT-MSC- and UC-MSC-treated pigs, and the defects were filled primarily with hyaline cartilage. In AT-MSC-EV- and UC-MSC-EV-treated pigs, a partial cartilage-bone tissue repair was observed, and the defects were filled primarily with fibrocartilage. The control pigs demonstrated limited regeneration capacity. The microcomputed tomography parameters of the subchondral bone indicated the ongoing progression of OA in controls, whereas in the MSC- and MSC-EV-treated pigs, the parameters indicated the cessation of OA progression. Moreover, no serious side effects were observed after the administration of products containing MSCs or MSC-EVs. The results indicate the safety and regenerative activity of MSCs on injured tissues, which favors not only the healing and improvement of bone structure but also the formation of hyaline cartilage. Superior tissue repair was observed after the administration of products containing AT-MSCs. The treatment of OA with MSC-EVs needs further standardization.

**Graphical Abstract:**

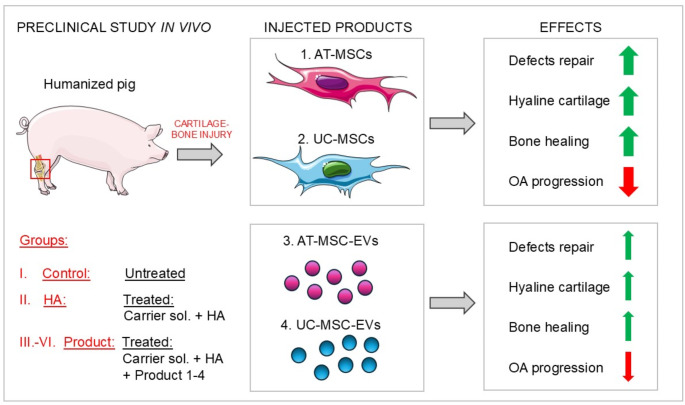

**Supplementary Information:**

The online version contains supplementary material available at 10.1007/s12015-025-10853-4.

## Introduction

Diseases related to articular cartilage damage are currently among the leading causes of disability worldwide [1]. One such condition, osteoarthritis (OA), is a degenerative joint condition that causes pain, swelling, and stiffness, affecting the ability of a person to move freely. OA affects the entire joint, including the articular cartilage, resulting in a narrowing of the joint space and an increase in direct loading of the underlying bone, which may cause loss of joint mobility. Progressive cartilage erosion is accompanied by structural changes in the underlying subchondral bone, low-grade inflammation, and synovitis [2]. As OA is more prevalent in people older than 55 y (mostly women), the global prevalence is expected to increase with increasing population age. The typical onset of OA is in the late 40s to mid-50s, although OA may also affect younger people, including athletes and people who sustain joint injury or trauma or suffer from rheumatoid arthritis, gout, diabetes, or obesity [3]. OA is most common in the knees, hips, spine, and hands. According to the WHO report, it is predicted that by 2050, OA will affect 130 million people worldwide, of which approximately 40 million will be severely disabled [1].

It is well known that there is no cure for OA that is able to recreate the degraded articular cartilage to eliminate the primary symptoms. However, according to the OA guidelines and a systematic review of the OA literature, worldwide experts from relevant medical disciplines have recommended treatment modalities for patients suffering from knee OA. After pain is managed, the subsequent core interventions for patients with OA, with or without comorbidities, include biomechanical interventions, intraarticular corticosteroids, exercise, self-management and education, strength training and weight management [4]. It has been shown that intraarticular injections of corticosteroids improve function in patients with OA but provide only short-term pain relief (1–2 weeks in randomized controlled trials). Moreover, intraarticular injections administered more frequently than once every four months may result in additional cartilage and joint damage [5, 6]. In recent years, the injection of hyaluronic acid (HA) has become a more popular treatment option for OA because of its high tolerability, improved joint lubrication and ability to delay the need for prosthetic surgery [7]. A similar beneficial effect was observed following the injection of platelet-rich plasma (PRP), which effectively decreased pain and stiffness and improved functional joint motion in patients with OA [8]. Injection of PRP provides continued pain relief for up to 1 y after injection, whereas the administration of corticosteroids and HA have good efficacy and are suitable for many patients but lack this longevity [9]. The most common surgical intervention for end-stage knee OA is total knee arthroplasty, an invasive procedure that is often associated with the risk of complications and should be performed as a last resort after exhausting all other therapeutic options [10].

Due to the lack of an effective and safe procedure for the treatment of OA, which could effectively eliminate not only symptoms but also their underlying causes, new innovative therapeutic approaches, including those that use stem cells, are needed [11]. Mesenchymal stem/stromal cells (MSCs), a specific type of adult stem cells, possess high potential in tissue engineering and regenerative therapy due to their capacity for self-renewal and great differentiation potential. Their wide availability, multipotent characteristics, and low immunogenicity have made them a promising treatment alternative in the field of regenerative medicine [11]. MSCs are able to efficiently downregulate immune inflammatory processes, making them promising candidates for inhibiting the influx of immune cells characteristic of OA [11]. Although the precise mechanism through which MSCs promote joint repair is not yet known, it is postulated that implanted MSCs differentiate into chondrocytes and may also secrete bioactive derivatives (such as extracellular vesicles (EVs)) that encourage the proliferation and anabolism of articular chondrocytes [12]. Therefore, the objective of the present study was to compare the safety and pro-regenerative potential of MSCs derived from two independent source tissues, human adipose tissue (AT) and human umbilical cord (UC), and their EV derivatives to select the most optimal biologically active substance for the development of advanced therapy medicinal products (ATMPs) for further clinical trials in humans. In this study, we meticulously evaluated the condition of the articular cartilage, subchondral bone plate, and trabecular bone and identified the types of newly formed tissues in cartilage-bone defects after intraarticular injection of investigational products. Preclinical tests of investigational medicinal products in in vivo models are required in order to obtain approval to conduct clinical trials in humans.

## Methods

### Isolation and Culture of AT-MSCs

Human adipose tissue (AT) was collected from healthy donors via liposuction with the approval of the Bioethics Committee of the Regional Medical Chamber in Krakow, Poland (Opinion No. 122.6120.13.2017). The collected lipoaspirate was washed with PBS solution without Ca^2+^ or Mg^2+^ (HyClone, GE Healthcare Life Sciences) supplemented with 1% Antibiotic-Antimycotic (Thermo Fisher Scientific) to prevent accidental microbial/fungal contamination and remove red blood cells. Stromal vascular fraction (SVF) cells were isolated via the enzymatic digestion of AT with 0.2 PZ U/mg Collagenase NB 6 GMP Grade (Nordmark) for 40 min at 37 °C. The activity of the collagenase was stopped by cooling the tubes with AT during further centrifugation (370× *g*, 10 min, 17 °C). After centrifugation, the pellet containing the SVF cells was resuspended in PBS and passed through a 100-µm strainer (Corning) to remove the remaining veins or tissue debris. The strainer was washed with additional PBS solution without Ca^2+^ or Mg^2+^ (HyClone), and the tube containing the cells was centrifuged (350× *g*, 7 min, RT). The pellet was subsequently resuspended in PBS without Ca^2+^ or Mg^2+^ (HyClone), and the total number of isolated cells in the Bürker chamber was counted. Finally, the SVF suspension was seeded in culture flasks (Corning) in complete cell culture medium (αMEM) supplemented with 10% human platelet lysate MultiPL’30 (hPL; both from Macopharma), 2 IU/mL Heparinum WZF (Polfa S.A.), and 1% Antibiotic-Antimycotic (Thermo Fisher Scientific) and further cultured under standard culture conditions (37 °C, 5% CO_2_, 95% humidity). At 48 h post-seeding, nonadherent cells were removed, and the adherent fraction of the SVF containing AT-derived mesenchymal stem/stromal cells (AT-MSCs) was further cultured. AT-MSCs were passaged with 1X TrypLE Select Enzyme (Thermo Fisher Scientific) when the cell confluence reached approximately 80–90% (after approximately 4–5 d of culture). Cells from the 1st passage were cultivated in αMEM supplemented with 10% human platelet lysate MultiPL’30 (both from Macopharma), 2 IU/mL Heparinum WZF (Polfa S.A.), 100 IU/mL penicillin, and 10 µg/mL streptomycin (Thermo Fisher Scientific). The morphology and confluence of AT-MSCs were evaluated using an Olympus IX81 microscope equipped with a MicroPublisher 3.3 RTV camera (Olympus).

### Isolation and Culture of UC-MSCs

Umbilical cord-derived mesenchymal stem/stromal cells (UC-MSCs) were isolated from human umbilical cords *via* the explant method following mechanical digestion. The 15–20-cm-long tissue samples were provided by Polish Stem Cells Bank S.A. (Poland), who was a partner in the STRATEGMED III project. The umbilical cord was washed with PBS without Ca^2+^ or Mg^2+^ (HyClone) supplemented with 1% Antibiotic-Antimycotic (Thermo Fisher Scientific). Subsequently, the blood vessels were removed, and the entire remaining umbilical cord tissue was cut into small pieces that were transferred to TC-treated culture dishes with a diameter (Ø) equal to 10 cm (Eppendorf). The cells were cultured in DMEM/F12 medium (Merck) supplemented with 10% fetal bovine serum (FBS, Merck), 100 IU/mL penicillin, and 10 µg/mL streptomycin (Thermo Fisher Scientific) under standard culture conditions (37 °C, 5% CO_2_, 95% humidity) to allow the migration of cells from explants. After 5 d, the tissue explants were removed, and the medium was changed. The medium was subsequently changed every 3–4 d. When the cell confluence reached approximately 70–80%, cells were harvested using 1X TrypLE Select Enzyme (Thermo Fisher Scientific) and prepared for further propagation or experiments. The morphology and confluence of the UC-MSCs were evaluated using an Olympus IX81 microscope equipped with a MicroPublisher 3.3 RTV camera (Olympus).

### Antigenic Phenotyping of MSCs by Flow Cytometry

To confirm the identity of isolated AT-MSCs and UC-MSCs according to the minimal criteria for definition of multipotent MSCs published by the ISCT [13], cells from the 3rd passage were resuspended in standard staining medium (αMEM supplemented with 2% hPL and 2 IU/mL Heparinum WZF for AT-MSCs or DMEM/F12 supplemented with 2% FBS for UC-MSCs) and further immunolabeled with the following monoclonal antibodies: anti-CD14 (FITC, clone: MφP9, BD Biosciences), anti-CD19 (FITC, clone: HIB19, BD Biosciences), anti-CD34 (PE or APC, clone: 581, BD Biosciences), anti-CD45 (PE or FITC, clone: HI30, BioLegend), anti-HLA-DR (PE or APC, clone: L243, BioLegend), anti-CD73 (PE or APC, clone: AD2, BioLegend), anti-CD90 (PE or APC, clone: 5E10, BioLegend), and anti-CD105 (PE or APC, clone: 43A3, BioLegend) according to the manufacturer’s protocols for 30 min at 4 °C. Cells were washed with PBS without Ca^2+^ or Mg^2+^ (HyClone) and analyzed using a BD LSRFortessa Flow Cytometer and FACS Diva software (Becton Dickinson).

### Adipogenic, Chondrogenic and Osteogenic Differentiation of MSCs

#### AT-MSCs

For osteogenic and adipogenic differentiation, 5.0 × 10^3^ or 1.0 × 10^4^ AT-MSCs, respectively, were seeded per 1 cm^2^ in complete cell culture medium (αMEM supplemented with 10% hPL, 2 IU/mL Heparinum WZF, 100 IU/mL penicillin, and 10 µg/mL streptomycin) and further cultured until approximately 60% confluence. The culture medium was replaced with StemPro Osteogenesis Differentiation Medium or StemPro Adipogenesis Differentiation Medium (Thermo Fisher Scientific), which stimulate osteogenic or adipogenic differentiation, respectively. To induce chondrogenic differentiation, AT-MSC micromass cultures were generated by seeding 5-µL droplets of cell suspension (1.6 × 10^7^ viable cells/mL) and incubating them for 1 h under high-humidity conditions; further micromasses were flooded with complete culture medium. The medium was changed to StemPro Chondrogenesis Differentiation Medium (Thermo Fisher Scientific) after 24 h. The differentiation media were changed every 2–3 d. The cells were examined for adipogenic differentiation on the 11th day of differentiation by direct microscopic observation to confirm the presence of oil droplets characteristic of adipogenesis. For chondrogenic and osteogenic differentiation, the cells were examined after 14 or 21 d of culture, respectively, after histological staining (with alizarin red or alcian blue according to the manufacturer’s protocols; Merck) to identify the cell phenotype.

#### UC-MSCs

UC-MSCs were seeded on a 6-well plate (Eppendorf) at a density of 4 × 10^4^ per well and cultured in StemPro Osteogenesis Differentiation Medium, StemPro Adipogenesis Differentiation Medium or StemPro Chondrogenesis Differentiation Medium (Thermo Fisher Scientific) to stimulate osteogenic, adipogenic, or chondrogenic differentiation. The differentiation media were changed every 2–3 d. After 21 d of differentiation, to evaluate the ability of the cells to differentiate into cells of mesodermal origin, the UC-MSCs were stained with alizarin red, oil red O, or alcian blue (Merck) according to the manufacturer’s protocols.

### EV Isolation

For the isolation of EVs from UC-MSCs and AT-MSCs, cells from the 5th − 7th passages were seeded and cultured in ultracentrifuged media to eliminate EVs and other small particles of FBS or hPL origin. All media were ultracentrifuged (100 000× *g*, 18 h, 4 °C) using an Optima XPN-90 ultracentrifuge with a Type 50.2 Ti fixed-angle rotor (Beckman Coulter). The media supernatants were then used for cell culture. Conditioned media (CM) were collected from confluent cultures, and the media were replaced 3 d before CM collection. All CM were stored at −80 °C until further processing. EVs were subsequently isolated from CM according to previously described methods [14]. Briefly, CM were centrifuged to remove cell debris (2000× g, 20 min, 4 °C). Next, the supernatants were ultracentrifuged (100 000× g, 70 min, 4 °C) using an Optima XPN-90 ultracentrifuge with a Type 50.2 Ti fixed-angle rotor. EV pellets were washed in 0.2-µm-filtered PBS without Ca^2+^ or Mg^2+^ (Lonza) and ultracentrifuged again with the same parameters. The obtained pellets containing EVs were resuspended in 0.2-µm-filtered PBS without Ca^2+^ or Mg^2+^. The samples were stored at −80 °C for further analysis.

## Nanoparticle Tracking Analysis (NTA)

To evaluate the size distribution of the EV samples, a NanoSight NS300 analyzer (Malvern Panalytical) was used. For the measurement, EV samples were 1000X diluted in 0.2-µm-filtered PBS without Ca^2+^ or Mg^2+^ to a final volume of 3 ml. Each sample tracking was performed with three repetitions, with camera level 13 and the threshold parameter set at 2. The average size of the particles was calculated using NTA Software ver. 3.4 (Malvern Panalytical).

### High-Resolution Flow Cytometry

EV samples were stained with one of the following APC-conjugated mouse monoclonal antibodies: anti-CD34 (clone: 581; BD Bioscience), anti-CD63 (clone: MEM-259; Thermo Fisher Scientific), anti-CD45 (clone: HI30), anti-CD73 (clone: AD2), anti-CD81 (clone: 5A6), anti-CD90 (clone: 5E10), anti-CD105 (clone: 43A3) or an appropriate isotype-matched control (all from BioLegend). Prior to staining, appropriate antibodies were resuspended in 0.2-µm-filtered PBS without Ca^2+^ or Mg^2+^ and centrifuged at 21 000× *g* for 20 min at 4 °C to remove potential debris and protein aggregates. Next, the supernatants were transferred into fresh tubes, and the EV samples were added and incubated for 30 min at 4 °C. Flow cytometry analysis was performed using an Apogee A60-Micro-PLUS cytometer and histogram software (Apogee Flow Systems, Hemel Hempstead, UK).

### Preparation of MSC Samples

After 3 d of culture under standard conditions, AT-MSCs and UC-MSCs (in the logarithmic phase of cell growth) were harvested using TrypLE Select Enzyme (Thermo Fisher Scientific) and counted using a Scepter Cell Counter (Merck). Then, 20 × 10^6^ MSCs of each type were aliquoted into 5 × 10^6^ cells in 1 ml appropriate complete culture medium and transported on ice to the Research Institute. The cells were gently mixed every 30 min. Each transplant specimen was individually prepared immediately prior to transplantation into pigs. The MSCs were centrifuged at 200× *g* for 6 min. The cell pellet was resuspended in 150 µl 0.9% NaCl supplemented with 10% human serum albumin (HSA) and pooled into one vial. Then, 1 ml hyaluronic acid (HA, DUROLANE, Bioventus LLC) was added to a sterile Eppendorf-like tube. The remaining volume of HA was mixed with the MSCs by discharging and drawing the suspension four times, avoiding air bubbles. Then, the syringe was filled with the previously aliquoted 1 ml HA and immediately transplanted into the knee joint cavity. For mixing, a syringe from the DUROLANE Kit and Ø1.2 × 40 mm needle (KD FINE) were used. The viability of the MSCs was checked before and after mixing with HA by trypan blue staining.

### Preparation of MSC-EV Samples

AT-MSC-EVs and UC-MSC-EVs resuspended in 0.2-µm-filtered PBS without Ca^2+^ and Mg^2+^ (Lonza) were transported on ice to the Research Institute. The AT-MSC-EV suspensions containing 2.0 × 10^11^ particles and UC-MSC-EV suspensions containing 2.5 × 10^11^ particles were diluted to a final volume of 150 µl in 0.9% NaCl supplemented with 10% human serum albumin (HSA) and subsequently mixed with HA as previously described. The prepared specimens were immediately transplanted into the knee joint cavity.

### Animals

All procedures related to the use of animals were performed at the Experimental Station Żerniki Wielkie LLC of the National Research Institute of Animal Production, in accordance with the approval of the 2nd Local Institutional Animal Care and Use Committee (IACUC) in Krakow, Poland (Approval number: 39/2016). In the present study, transgenic pigs (male, female) with inactivated ULBP1 (pig line ULBP1) or GGTA1 (pig line ZFN) genes were used. Pigs of each line were obtained by crossbreeding of previously characterized individuals from the F0 generation, which were produced using CRISPR/Cas9 technology to disrupt porcine genes [15]. In the experiment, only piglets with confirmed inactivation of the ULBP1 or GGTA1 gene were used, and to minimize potential confounders, only pigs that had a body weight (BW) of approximately 60 kg and were in good health without motor disorders were used. This in vivo study was performed in accordance with the ARRIVE guidelines 2.0.

### Pig Line ULBP1

Activated NK cells are capable of the cytotoxic destruction of endothelial cells. Introduction of a gene encoding human leukocyte antigen (HLA), a ligand of the NKG2A receptor CD94/NKG2A, into endothelial cells partially protects porcine endothelial cells from destruction in vitro [16]. The cytotoxic effect on porcine endothelial cells, leading to their lysis, is also caused by the binding of the porcine ULBP1 antigen to NKG2D receptors and the previously unidentified NKp44 antigen. Surface antigens present on the surface of cells other than endothelial cells may also be involved in NK cell activation. Nevertheless, the elimination of ULBP1 from the surface of endothelial cells and the expression of human HLA-E may be effective in preventing NK-cell-mediated xenograft rejection [17]. CRISPR/Cas9 technology was used to disrupt the porcine ULBP1 gene. The design of two sgRNAs targeting the region of exon 2 of the pULBP1 gene has been described previously [17]. Genomic DNA was extracted from ear biopsy samples from potentially genetically modified pigs using a Kapa Express Extract DNA Extraction Kit with PCR ReadyMix (Kapa Biosystems). PCR was performed using the ULBP1-F (5′-CTC ACC TGC GTT TTG CCT TC-3′) and ULBP1-R (5′-CCT TGA GGA AGT CCC CAA CG-3′) primers under the following conditions: 94 °C for 180 s, followed by 35 cycles of 94 °C for 30 s, 58 °C for 45 s, and 72 °C for 30 s and a final extension at 72 °C for 360 s. The 246-bp PCR product was directly sequenced using an automated genetic analyzer (Applied Biosystems Prism) with the ULBP1-R primer.

### Pig Line ZFN

The basis for xenograft rejection is the Gal antigen (Galα1.3Gal) present on the glycolipids and glycoproteins found on the surface of the donor cells. The Gal antigen is generated by attaching the galactose molecule to N-acetyllactosamine through a α1,3-glycosidic bond via catalysis from the α1,3-galactosyltransferase enzyme (GGTA1, α1.3GT). Both the enzyme and the sugar residue are absent in humans and Old World monkeys. In humans, however, xenoreactive antibodies against the porcine Gal antigen are present; these antibodies recognize the Gal epitope on the surface of porcine endothelial cells, among others, and activate the enzymatic complement cascade in the recipient, which results in rapid rejection of the xenograft. In the cells of transgenic animals with an inactivated GGTA1 gene, no enzyme is present; thus, no Gal epitope is formed. The organs of such animals are characterized by reduced immunogenicity. The pZFN1 and pZFN2 plasmids encoding zinc finger nucleases targeting the exon 9 region of the GGTA1 gene encoding the catalytic domain of the enzyme have been described previously [15]. Genomic DNA was extracted from ear biopsy samples from potentially genetically modified pigs. PCR was performed using ZFN-F (5’-TGCGTTCCTTTAAAGTGTTTGA-3’) and ZFN-R (5’-CTGTAGCTGAGCCACCGACT-3’) primers under the following conditions: 94 °C for 3 min, followed by 35 cycles of 94 °C for 30 s, 58 °C for 45 s, and 72 °C for 30 s and a final extension at 72 °C for 6 min. The 200-bp PCR product was sequenced using an automated genetic analyzer (Applied Biosystems Prism) with the ZFN-R primer.

### Large Preclinical Pig Model of Cartilage-Bone Injury In Vivo

**Fig. 1:**
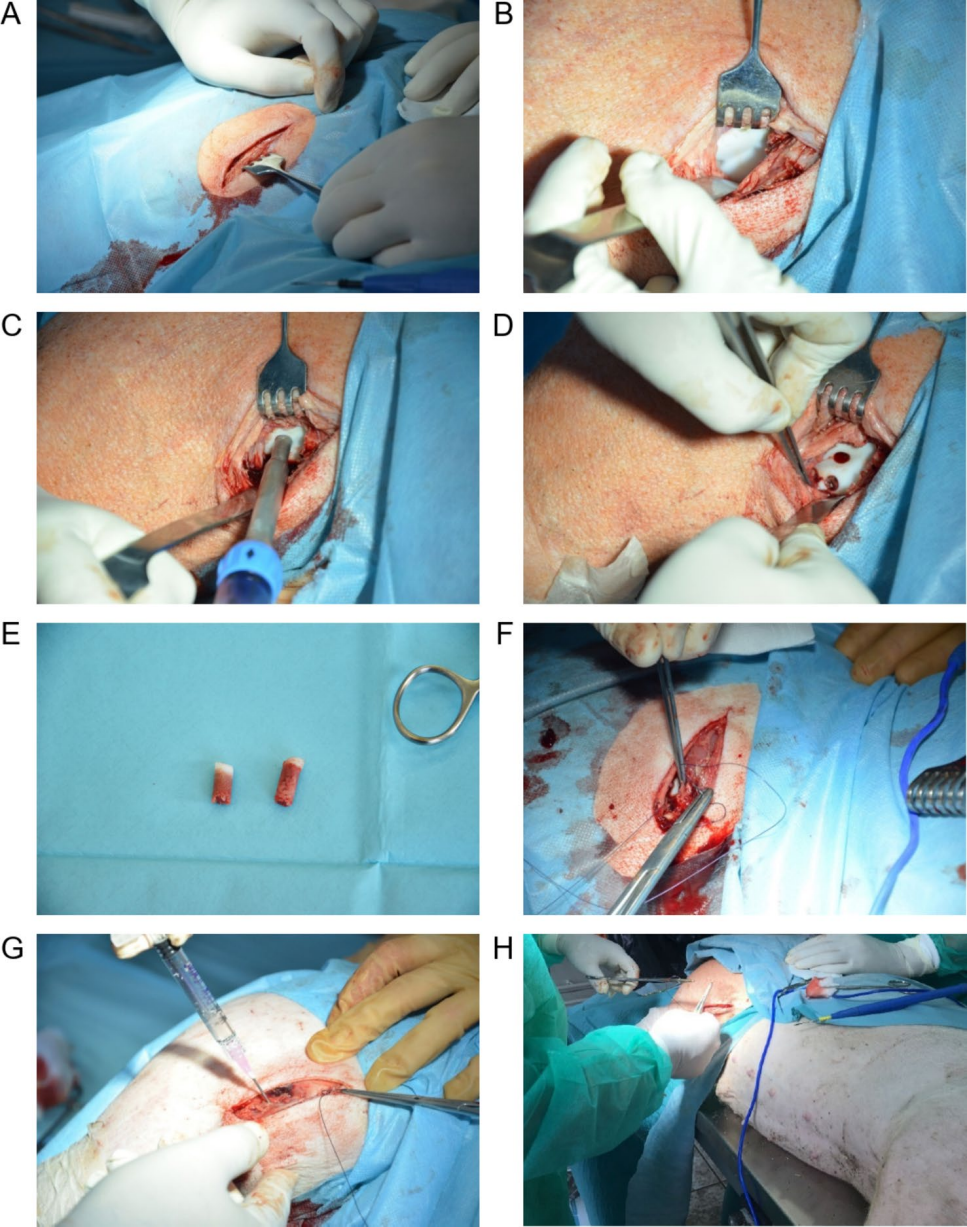
Scheme of the surgical procedure for inducing mechanical cartilage-bone injury in the femoral bone in the knee. **A** Skin incision at the surgical site. **B** Opening of the knee joint. Exposure of articular surfaces (containing the condyles of the femur). **C** Inducing mechanical injury (cavities) in the articular cartilage and subcartilage tissues. **D** Exposed cavities (defects) in the lateral and medial condyles of the femur. **E** Pieces of removed cartilage and subcartilage tissues. **F** Closing of the joint capsule. **G** Intraarticular administration of the investigational product. **H** Placing stitches at the skin incision site

In the present study, a transgenic pig model in which cartilage-bone injury was mechanically induced was used, as shown in Fig. 1. The pigs were pretreated with 2.0 mg/kg BW azaperone (Stresnil, Janssen Pharmaceutica). General anesthesia was subsequently induced by 10.0 mg/kg BW ketamine (Ketamina 10%, Biowet Pulawy) and azaperone 1.00 mg/kg BW. Anesthesia was supplemented with local anesthesia of the knee with 10–12 ml 5% polocaine (Polocaina Hydrochloride 5%, Biowet Pulawy). Briefly, after opening the knee, in the middle of the surface of the lateral and medial condyles of the femoral bone, tissue defects (one defect/condyle) with a diameter of 6 mm and a depth of 10 mm were made using the COR Disposable Kit (6 mm with perpendicularity (DePuy Mitek & Johnson&Johnson Company). Each defect comprised a complete cross section of the articular cartilage tissue and the subcortical tissue of the condyle of the femur. The joint capsule was closed using absorbable sutures (PGA 2/0, YAVO). The prepared product was subsequently injected immediately into the knee joint cavity, after which stitches were placed at the incision site using absorbable sutures (PGA 2/0, YAVO).

The experimental groups were as follows: cartilage-bone defects were left untreated for self-healing (group: Control; *N* = 6); cartilage-bone defects were treated with investigational products containing as an active substance AT-MSCs (group: AT-MSCs; *N* = 7), UC-MSCs (group: UC-MSCs; *N* = 6), AT-MSC-EVs (group: AT-MSC-EVs; *N* = 5), or UC-MSC-EVs (group: UC-MSC-EVs; *N* = 5, with 4 pigs surviving) resuspended in carrier solution and mixed with hyaluronic acid (HA) prior to intraarticular administration, or carrier solution mixed with HA was applied for comparison (group: HA; *N* = 6, with tissue collection was possible in only 5 pigs due to stiffness and fusion of the knee). In the experiment, 35 pigs were randomly (using randomization envelopes) assigned to the abovementioned groups. All the investigators involved in the in vivo study were unaware of the allocation of the pigs to groups during the entire experiment. The only person who was aware of the allocation of pigs to groups was the assistant who conducted randomization and prepared products for intraarticular administration. Each pig was considered to be a single experimental unit, and pigs were kept in separate sties (within one piggery) throughout the experiment to minimize potential confounders.

After surgery, a 10-d course of antibiotics and analgesics was administered: SHOTAPEN 0.5–1.0 ml/10 kg BW (Virbac) and meloxicam 0.2–0.3 mg/kg BW (Metacam 5%, Boehringer Ingelheim Vetmedica GmbH). Animal welfare and health after surgery were subsequently monitored regularly over a 6-m follow-up period. At the end of the experiment, the pigs were euthanized to collect biological material for further examination of the healing process by microcomputed tomography and histological staining.

### Material Collection

After 6 m of follow-up, the pigs were sacrificed using sodium pentobarbital with an overdose of 20 mg/kg BW (Morbital, Biowet). The operated knee joints were dissected, and fragments of both femoral condyles (covering the places where the defects were made) were subsequently collected. To compare the degree of healing in the defect and possible internal changes in the region of the defect, identical bone fragments from the left unoperated knee were collected. The cartilage-bone samples were washed in PBS (HyClone) and fixed in 10% buffered formalin pH 7.4 (Alpinus Chemia). The collected tissues were labeled only with animal identification numbers and sent in such (blinded) form for further analyses.

### Microcomputed Tomography

The collected bone-cartilage samples were scanned using a high-resolution X-ray scanner, GE Phoenix v|tome|s m (GE, Germany). The X-ray settings were 200 kV and 300 µA, and a copper filter with a thickness of 0.1 mm was used to minimize beam hardening artifacts. The microcomputed tomography (micro-CT) images had a 3D reconstruction cubic voxel size of 25 μm. Tomograms were reconstructed using Phoenix Datos|x 2.0 reconstruction software (GE Sensing & Inspection Technologies GmbH, Germany). The reconstruction was based on the 2000 projections and carried out using 16-bit color depth. For the calibration of bone mineral density (BMD), the Micro-CT HA D25 Phantom (Æ = 25 mm, Æi = 5 mm) produced by QRM GmbH (Germany) was scanned together with each sample. The hydroxyapatite (HAp) phantom contained 5 cylinders of different densities of HAp crystals (0, 50, 200, 800 and 1200 mg HAp/cm^3^). Calibration curves were then established for each sample, correlating gray density (GD) values with HAp density (g HAp/cm^3^), enabling quantitative assessment of bone mineral density (BMD).

All image analyses were conducted using ImageJ 1.49b software, an open-source image processing tool developed by the US National Institute of Health in Bethesda, MD, USA [18]. The following parameters were assessed: volume of cartilage defects, volume of bone defects, cartilage thickness above healthy bone and bone defect, bone mineral density (BMD) and thickness of the subchondral bone plate, BMD and bone-to-total volume ratio (BV/TV) of the trabecular bone, presence of neoplasm events, and presence of potential side effects related to the injected product.

Bone microstructure parameters were calculated using the BoneJ plugin [19] within ImageJ software. To avoid time-consuming selection of the volume of interest (VOI) for cartilage, cartilage defects, and bone defects on each slice, the operator manually marked the approximated VOI every fifth slice, which was then interpolated onto the remaining slices (Supplementary File 1: Fig. S1). The volumes of cartilage and bone were independently assessed using manually selected and interpolated ROIs (Supplementary File 1: Fig. S1A, B). Cartilage thickness was evaluated in two locations: above the visible bone defect (Supplementary File 1: Fig. S1C) and next to the bone defect (Supplementary File 1: Fig. S1D). Measurements were also conducted at the subchondral bone plate (SBP) beneath unaffected cartilage (Supplementary File 1: Fig. S1E) and at the trabecular bone around the defect (Tb.D). The size of the Tb.D was determined by expanding the VOI of the bone defect by 1.5 mm in each direction (Supplementary File 1: Fig. S1F). In cases where a bone defect was absent in the sample, the VOI was selected beneath the SBP (with a square size of VOI of 5 × 5 × 5 mm). The following parameters were calculated: for the SBP, BMD and thickness, and for the Tb.D, BMD and BV/TV. The changes in the cartilage and bone for this study were visualized using Dragonfly software (Version 2023.1, Comet Technologies Canada Inc., Montreal, Canada). The analysis was conducted for the following numbers of units (pigs) per experimental group: Control (*N* = 4), AT-MSCs (*N* = 6), UC-MSCs (*N* = 6), AT-MSC-EVs (*N* = 5), UC-MSC-EVs (*N* = 4), and HA (*N* = 4). No unit was excluded from the analysis, but for technical reasons, the number of analyzed samples obtained from separate units was lower than the total number of pigs who completed the study. The data analysis was conducted for pigs in groups whose names were blinded until the statistical analysis was conducted. Unblinding was conducted while the summary of the data was prepared.

### Histology

The bone-cartilage samples collected for histological analysis preserved with 10% neutral buffered formalin were decalcified with 5% nitric acid (diluted in distilled water, ChemPUR), and 5-mm-thick cross sections were prepared. When a detailed examination revealed areas of tissue repair or cartilage erosion, only lesional sections were collected for further evaluation. In the absence of macroscopically detectable lesions, the whole material was assembled for histopathological examination. The qualified tissues were embedded in paraffin, cut into thin Sect. (3 μm thick) and stained with hematoxylin and eosin (H&E, Hematoxylin 3G Tissue-Tek & Eosin Tissue-Tek, Sakura) on a slide stainer (Tissue-Tek Prisma, Sakura). Slides were examined under an Olympus BX53 microscope, and images were captured with digital image acquisition software (cellSens Dimension, Olympus). The analysis was performed for all units (pigs) who completed the study. The analysis was conducted for pigs in groups whose names were blinded until the histological assessment was conducted. Unblinding was conducted after analysis while the summary of the data was prepared.

During the analysis, the following parameters were assessed: the presence of (i) thickening of the bone trabeculae, (ii) inflammatory infiltration, (iii) neoplasm events, and (iv) potential side effects related to the injected product; and the type of newly formed tissue filling the cartilage-bone defects.

### Statistical Analysis

The data are presented as the means ± SDs. Statistical analyses were performed via the Mann-Whitney U test (for data with a nonnormal distribution, as indicated by the Shapiro-Wilk test) or ANOVA (for data with a normal distribution, as indicated by the Shapiro-Wilk test) using the Prism software package 8.00 (GraphPad Software). *p* < 0.05 was considered to indicate statistical significance. Additional biostatistical consultation was performed to assess the optimal number of pigs per group (considering the 3R rules).

## Results

### Confirmation of AT-MSCs, UC-MSCs and Their Derivatives Identity

**Fig. 2:**
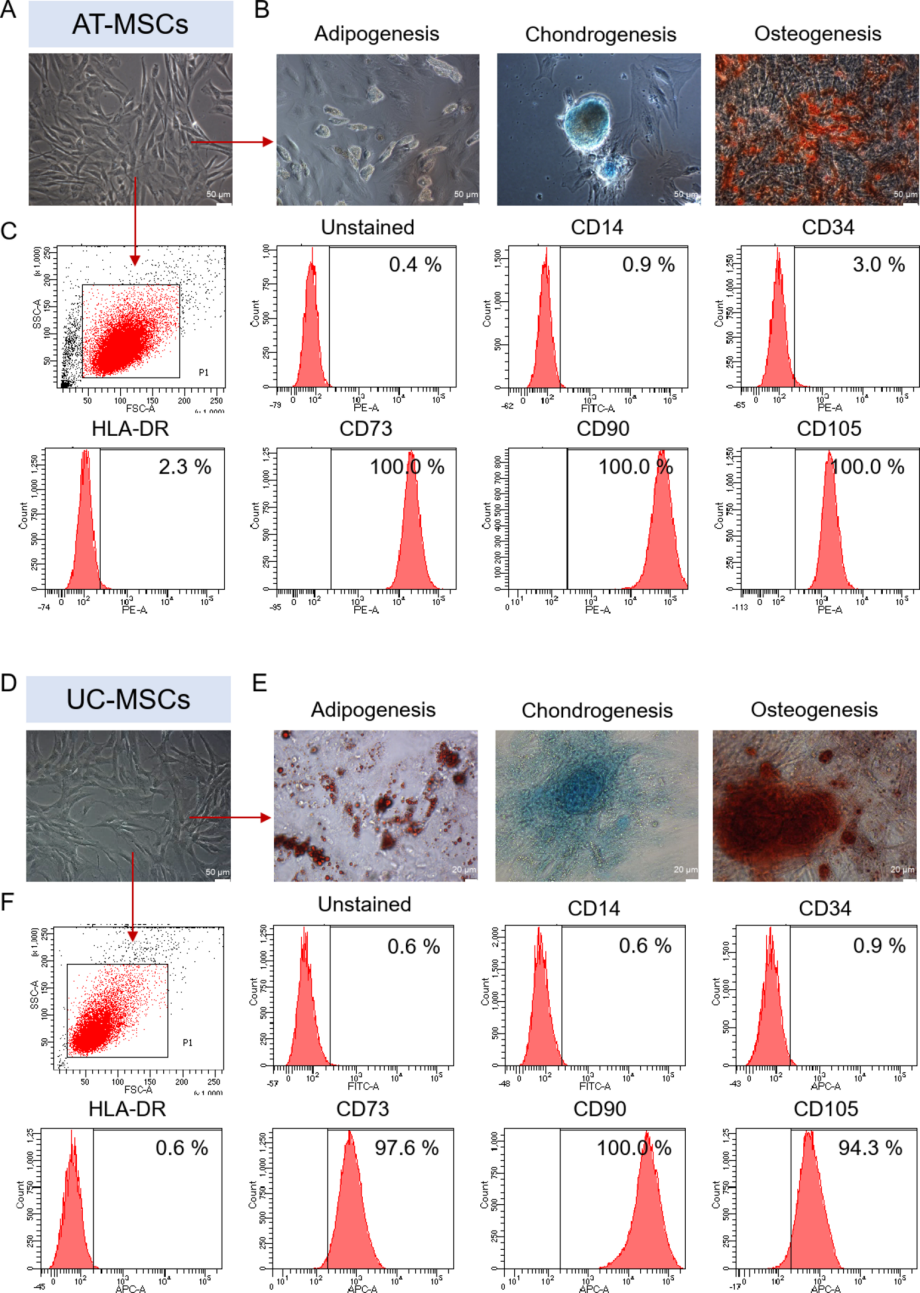
Characterization of MSCs according to the criteria defining multipotent MSCs recommended by the International Society for Cellular Therapy. **A** Representative image of the morphology of AT-MSCs. **B** Trilineage differentiation potential of AT-MSCs. Representative images of AT-MSCs differentiated into adipocytes, chondroblasts and osteoblasts. AT-MSCs were cultured using a StemPro Adipogenesis Differentiation Medium (for 11 d), StemPro Chondrogenesis Differentiation Medium (for 14 d) or StemPro Osteogenesis Differentiation Medium (for 21 d). AT-MSCs were subsequently fixed with paraformaldehyde and stained with alcian blue (blue staining of sulfated proteoglycans is characteristic of chondrogenic differentiation) or alizarin red S (red staining of calcium phosphate deposits is characteristic of osteogenic differentiation); oil droplets characteristic of adipogenic differentiation were observed in unfixed and unstained AT-MSCs. **C** Antigenic profile of AT-MSCs by flow cytometry. Representative histograms of the expression of MSC-negative markers (CD14 and CD34), MSC-positive markers (CD73, CD90, and CD105), and the HLA-DR antigen on AT-MSCs. **D** Representative image of the morphology of UC-MSCs. **E** Trilineage differentiation potential of UC-MSCs. Representative images of UC-MSCs differentiated into adipocytes, chondroblasts and osteoblasts. UC-MSCs were cultured using a StemPro Adipogenesis Differentiation Medium, StemPro Chondrogenesis Differentiation Medium or StemPro Osteogenesis Differentiation Medium for 21 d. UC-MSCs were subsequently fixed with paraformaldehyde and stained with alcian blue, alizarin red S or oil red O (brown staining of oil droplets is characteristic of adipogenic differentiation). **F** Antigenic profile of UC-MSCs by flow cytometry. Representative histograms of the expression of MSC-negative markers (CD14 and CD34), MSC-positive markers (CD73, CD90, and CD105), and the HLA-DR antigen on UC-MSCs. Scale bars: 50 μm–20 μm

In the present research, protocols for the isolation and culture of MSCs derived from two independent sources of human tissues were established: AT (obtained during a liposuction procedure from adult donors) and UC (representing fetal tissue). First, we evaluated the identity of both AT- (Fig. 2A-C) and UC- (Fig. 2D-F) derived cell populations as MSCs according to ISCT recommendations [13].

We found that AT-derived cells adhere to the polystyrene surfaces of cell culture flasks when maintained under standard culture conditions in vitro and are spindle-shaped cells with a fibroblast-like morphology (Fig. 2A). To confirm their trilineage differentiation potential and simultaneously determine their biological potential to transdifferentiate into cells that build body tissues (such as cartilage and bone), which is one of the possible mechanisms of MSC action in injured tissues, AT-derived cells were differentiated into chondroblasts, osteoblasts or adipocytes over a period of 14, 21, or 11 d (using tissue-specific differentiation media), respectively. Additionally, we found that AT-derived cells could be effectively differentiated into chondroblasts, osteoblasts and adipocytes in vitro (Fig. 2B). Using a flow cytometry platform, we subsequently demonstrated that our population of isolated AT-derived cells highly expressed MSC-specific markers such as CD73, CD90, and CD105 (> 98.0% of positive cells) and did not express markers of hematopoietic cells such as CD14, CD19, CD45, and CD34 or the HLA-DR antigen (< 2.0% of positive cells; Fig. 2C; Table 1). These results together confirmed the morphology, phenotype and potential for trilineage differentiation of isolated AT-derived MSCs (AT-MSCs) as defined by the minimal criteria recommended by the ISCT [13]. Similar investigation (according to ISCT recommendations [13]) of UC-derived cells revealed that the isolated cells could adhere to plastic surfaces, had trilineage differentiation potential, and had the characteristic antigenic profile (i.e., > 98.0% of positive cells for CD73, CD90, and CD105 along with < 2.0% of positive cells for CD14, CD19, CD45, and CD34 or the HLA-DR antigen), confirming their identity as UC-derived MSCs (UC-MSCs), representing a subpopulation of MSCs (Fig. 2D-F; Table 1).

**Table 1:** Antigenic phenotyping of the two MSC populations by flow cytometry

	AT-MSCs				UC-MSCs			
	Analysis				Analysis			
Antigen	*# 1*	*# 2*	*# 3*	Mean ± SD [%]	*# 1*	*# 2*	*# 3*	Mean ± SD [%]
CD14	0.9	0.4	0.3	**0.53 ± 0.26**	0.6	0.2	0.3	**0.37 ± 0.17**
CD19	0.3	0.0	0.3	**0.20 ± 0.17**	0.1	0.1	0.0	**0.07 ± 0.06**
CD34	3.0	0.6	0.6	**1.40 ± 1.13**	0.9	0.2	0.4	**0.50 ± 0.29**
CD45	0.8	0.8	0.8	**0.80 ± 0.00**	0.1	3.1	1.6	**1.60 ± 1.22**
HLA-DR	2.3	1.6	0.5	**1.47 ± 0.74**	0.6	0.6	4.7	**1.97 ± 1.93**
CD73	100.0	100.0	100.0	**100.00 ± 0.00**	97.6	100.0	100.0	**99.20 ± 1.13**
CD90	100.0	100.0	100.0	**100.00 ± 0.00**	100.0	100.0	100.0	**100.00 ± 0.00**
CD105	100.0	100.0	94.8	**98.27 ± 2.45**	94.3	100.0	100.0	**98.10 ± 2.69**

The values are presented as the percentage of positive cells for the analyzed marker

**Fig. 3:**
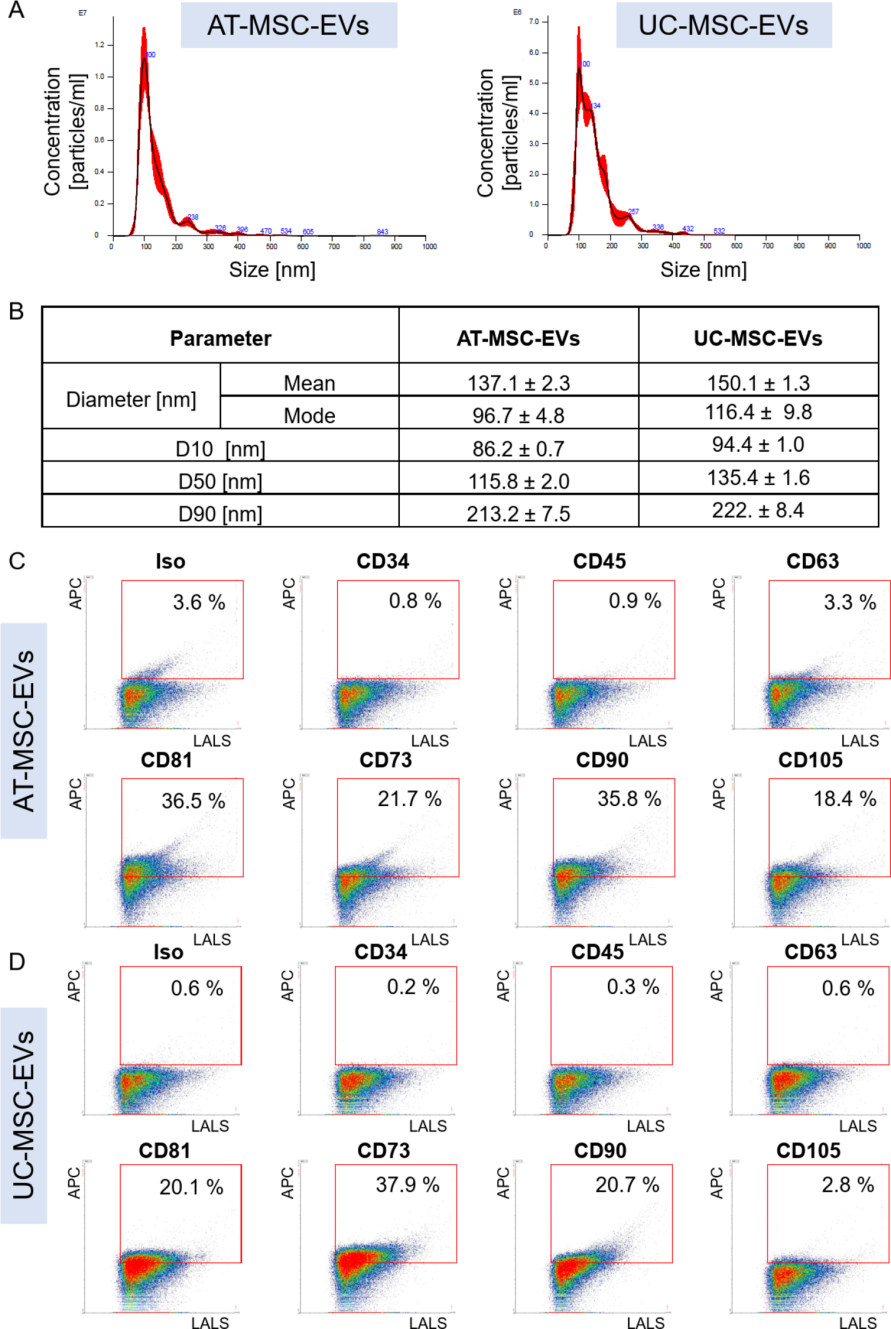
Characterization of AT-MSC-EVs and UC-MSC-EVs. **A** Representative histograms of the particle size distributions obtained by NTA analysis. **B** Summary of the particle size distribution data presented as the means ± SDs (*n* = 3 sample tracking repetitions). The D10, D50, and D90 parameters correspond to 10, 50 and 90% of the particles, respectively, that possess a diameter less than or equal to the given value. **C**,** D**. Phenotypes of AT-MSC-EVs and UC-MSC-EVs. The samples were analyzed via a high-resolution Apogee A60-Micro-PLUS flow cytometer. Representative dot plots of AT-MSC-EVs **C** and UC-MSC-EVs **D** stained with appropriate antibodies against selected markers. The percentage of particles positive for the analyzed marker is shown based on the autofluorescence of the unstained control. LALS represents large-angle light scatter parameter, which is proportional to the relative size of the particles; Iso – isotype control

To evaluate whether both MSC populations were capable of secreting some bioactive factors (which may contribute to the regeneration of injured tissues), the conditioned media harvested from AT-MSC and UC-MSC cultures were collected, and then the EVs were isolated using a sequential centrifugation method including a double ultracentrifugation step. After receiving our samples, we analyzed their identity following ISEV recommendations [20]. We found that isolates of both human AT- and UC-MSC-EVs represented heterogeneous populations of particles with sizes ranging from approximately 80–430 nm (Fig. 3A). The AT-MSC-EVs had a mean diameter of 137.1 ± 2.3 nm and were enriched in particles with a diameter of 96.7 ± 4.8 nm, whereas the UC-MSC-EVs had a mean diameter of 150.1 ± 1.3 nm and were enriched in particles with a diameter of 116.4 ± 9.8 nm (Fig. 3B). Flow cytometry analysis of our MSC-EV samples (derived from MSCs from both source tissues) revealed subpopulations of vesicles carrying parental cell-specific antigens (e.g., CD73, CD90, and CD105) and tetraspanins (e.g., CD63 and CD81), and in parallel, fewer than 1.0% of events were positive for hematopoietic cell markers (e.g., CD34 and CD45), as shown in Fig. 3C, D (values lower than 1.0% may correspond to nonspecific staining). Interestingly, the percentage of CD105-enriched EVs was higher in AT-MSC-EV samples than in UC-MSC-EV samples (Fig. 3C). Taken together, our results confirmed that our isolated samples contained AT- and UC-MSC-EVs according to ISEV recommendations for confirming the identity of EVs [20].

### Preclinical Evaluation of the Efficiency and Safety of Using AT-MSCs, UC-MSCs and Their Derivatives for the Treatment of Cartilage-Bone Injury in a Transgenic Pig Model

The main objective of this study was to preclinically evaluate the safety and biological potential (efficiency) of both MSC populations and their derivative EVs for the treatment of cartilage-bone injury in an in vivo pig model. Thus, transgenic pigs underwent surgical procedures to induce mechanical cartilage-bone injuries of the femoral bone in the knee (Fig. 1A-F), followed by injection of investigational products containing AT-MSCs, UC-MSCs, AT-MSC-EVs or UC-MSC-EVs (as an active substance) resuspended in carrier solution and further mixed with high-molecular-weight HA (Fig. 1G, H). Carrier solution mixed with high-molecular-weight HA was injected for comparison (group: HA). Pigs in these groups were compared with untreated pigs (cartilage-bone defects were left for self-healing; group: Control). The viability of both AT-MSCs and UC-MSCs was ≥ 97.0% before the preparation of the cell-based products but was ≥ 96.0% after mixing the cell suspensions with high-molecular-weight HA (Supplementary File 1: Fig. S2). The observed high viability of the MSCs used for the preparation of products ensures the proper biological properties of the MSCs and is crucial for the final outcome in pigs.

**Fig. 4:**
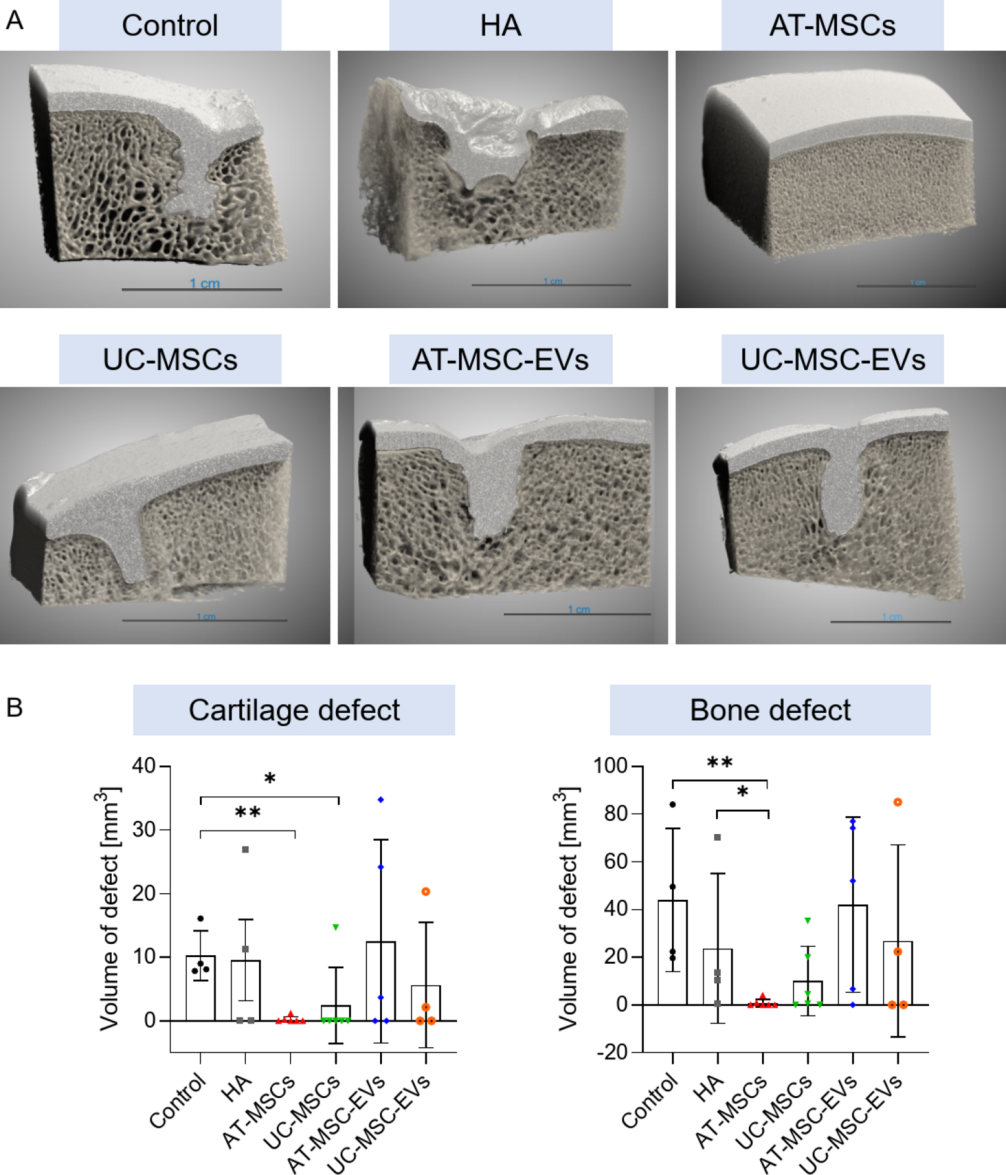
Evaluation of the pro-regenerative potential of AT-MSCs, UC-MSCs and their derivatives in a preclinical transgenic pig model of cartilage-bone injury. Transgenic pigs underwent surgical procedures to induce mechanical cartilage-bone injuries of the femoral bone in the knee and then either left to self-heal (Control) or followed by injection of HA (carrier solution mixed with HA), or with AT-MSCs, UC-MSCs, AT-MSC-EVs or UC-MSC-EVs resuspended in carrier solution and further mixed with HA. After a 6-m follow-up period, cartilage and bone samples from the femoral condyles were collected, fixed with buffered 10% formalin pH 7.4 and scanned using a high-resolution X-ray scanner. **A** Representative 3D visualizations of fragments of the femoral condyles (covering the places where the defects were made) in pigs in the experimental groups. **B** Quantitative analysis of cartilage and bone defects. The data obtained for each animal within a group are presented in graphs as indicated by the markers. *N* = 4‒6 pigs/group; Mann-Whitney U test, *p < 0.05, **p < 0.01

After 6 m of follow-up, microcomputed tomography revealed almost-fully-healed cartilage-bone defects mainly in two groups of pigs, the AT-MSCs and UC-MSCs groups, compared to pigs in the other groups, including the AT-MSC-EVs and UC-MSC-EVs groups (Fig. 4A). Importantly, cartilage and bone defects were almost never observed in pigs in the AT-MSCs group (Fig. 4B, Supplementary File 1: Tables S1 and S2), i.e., their volumes were 0.23 ± 0.47 mm^3^ for the cartilage defects and 0.81 ± 1.62 mm^3^ for the bone defects. The volumes of cartilage and bone defects were also low in pigs in the UC-MSCs group (2.45 ± 6.01 mm^3^ for cartilage defects and 10.10 ± 14.57 mm^3^ for bone defects; Fig. 4B). Interestingly, pigs in the HA group demonstrated limited regeneration capacity, i.e., tissue defects were still detected on the surface of the joints; moreover, the volume of cartilage defects was comparable to that of the defects in pigs in the Control group (9.54 ± 12.73 mm^3^ in the HA group vs. 10.26 ± 3.93 mm^3^ in the Control group), whereas the volume of bone defects was smaller than that in pigs in the Control group (23.72 ± 31.46 mm^3^ in the HA group vs. 43.92 mm^3^ ± 29.98 mm^3^ in the Control group), suggesting that treatment without biologically active MSCs or EVs was not sufficient to repair the defects and regenerate damaged tissues. Furthermore, we did not observe a high degree of cartilage-bone tissue regeneration in any of the pigs in the AT-MSC-EVs and UC-MSC-EVs groups (Fig. 4B).

**Fig. 5:**
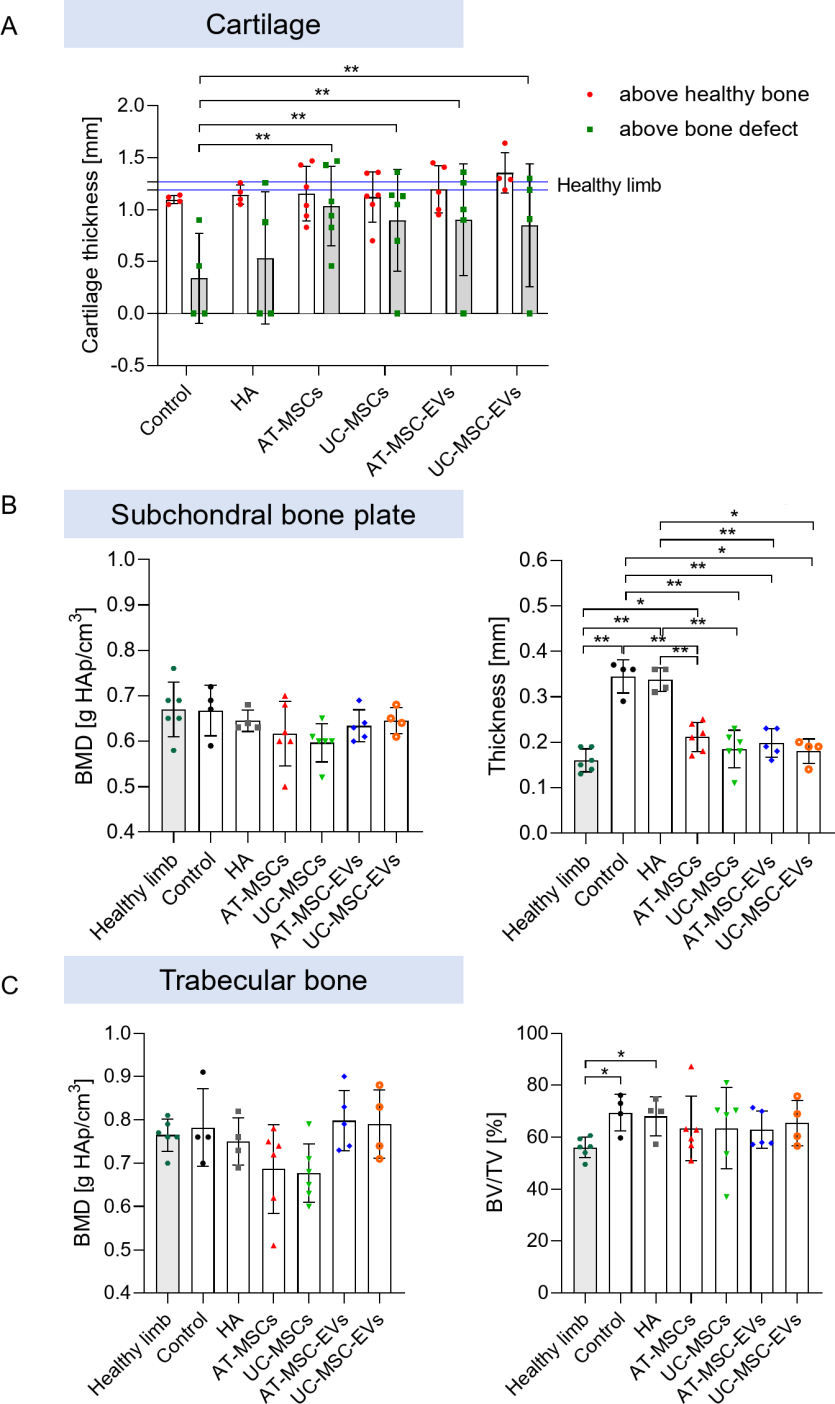
Quantification of the pro-regenerative potential of AT-MSCs, UC-MSCs and their derivatives in a preclinical transgenic pig model of cartilage-bone injury. The collected cartilage-bone samples were scanned using a high-resolution X-ray scanner, and the obtained images were subsequently analyzed using ImageJ 1.49b software. **A** Quantification of cartilage thickness above healthy bone and bone defects. For statistical analysis, two-way ANOVA was used. **B** Quantification of the bone mineral density (BMD) and thickness of the subchondral bone plate. For statistical analysis, ANOVA (for BMD) or the Mann-Whitney U test (for cartilage thickness analysis) was used. **C** Quantification of bone mineral density (BMD) and the bone-to-total volume ratio (BV/TV). For statistical analysis, one-way ANOVA (for BMD) or the Mann-Whitney U test (for BV/TV) was used. The data obtained for each animal within a group are presented in graphs as indicated by the markers. *N* = 4–6 pigs/group; *p < 0.05, **p < 0.01

Next, we analyzed the cartilage thickness above the bone defects and compared it with the cartilage thickness above healthy bone (Fig. 5A; Supplementary File 1: Tables S3 and S4). The cartilage thickness measured for the healthy untreated joint was comparable to the cartilage thickness above healthy bone in the operated (injured) joint. Interestingly, the lowest cartilage thickness (measured above a bone defect) was observed in pigs in the Control and HA groups, and this value was much lower than that for healthy cartilage (which was equal to approximately half the thickness of healthy cartilage). The greatest cartilage thickness above a bone defect was observed in pigs in the AT-MSCs, UC-MSCs, AT-MSC-EVs and UC-MSC-EVs groups. However, in pigs in the AT-MSCs group, similar cartilage thicknesses were observed above the bone defects and healthy bone (1.04 ± 0.38 and 1.16 ± 0.26 mm, respectively), indicating the superior pro-regenerative potential of AT-MSCs. Notably, treatment of cartilage-bone defects with biologically active products (such as MSCs or EVs) induced a better healing process, i.e., the formation of new cartilage above the bone defect, than that observed in pigs in the Control and HA groups (Fig. 5A).

In the present study, in addition to the articular cartilage condition, we also analyzed selected parameters of the subchondral bone, which was separated into two distinct anatomic entities: the subchondral bone plate (SBP) and the trabecular bone. The bone mineral density (BMD) of the SBP was slightly greater in pigs in the Control and HA groups than in pigs in the other groups (Fig. 5B; Supplementary File 1: Tables S5 and S6). Interestingly, patients with OA have been shown to have a 15% higher bone density than healthy controls [3]. The other parameter analyzed, SBP thickness, was also significantly greater in pigs in the Control and HA groups than in pigs in the other groups and in their healthy unoperated joints. Importantly, the thickness of the SBP in healthy unoperated joints was close to that measured in the operated joints of pigs in the AT-MSCs, UC-MSCs, AT-MSC-EVs, and UC-MSC-EVs groups (Fig. 5B), which may indicate the cessation of OA progression and continued tissue regeneration. In early OA, when the cartilage is still intact, the thickness of the subchondral cortical (bone) plate decreases due to an elevated rate of bone remodeling, whereas in late OA, when degenerative changes are evident in the articular cartilage, the thickness of the subchondral plate increases [21], which is consistent with the observations of our study.

With respect to the BMD of the trabecular bone, we observed slightly greater values in pigs in the Control and HA groups than in pigs in the AT-MSCs and UC-MSCs groups. Interestingly, the BMD values of the SBP and trabecular bone in healthy unoperated joint were close to the values obtained for the pigs in the Control and HA groups, which may have been caused by unloading of the operated limbs and shifting the body weight to the healthy leg. Moreover, degenerative changes in the articular cartilage of unoperated limbs were observed (data not shown). However, the other parameter, the ratio of bone volume to total sample volume (BV/TV) measured close to the site of the bone defect, was significantly greater in pigs in the Control and HA groups than in the unoperated joints and in pigs in the other groups (Fig. 5C; Supplementary File 1: Tables S7 and S8), which may indicate increased bone remodeling and formation around the defect. Patients with OA have been shown to have a 30% increase in bone volume [3]. Notably, histological analysis revealed thickening of the bone trabeculae in the subchondral layer in pigs in every group (Table 2), suggesting an ongoing postinjury bone remodeling process. However, the percentage of pigs that showed thickening of the bone trabeculae was the highest among pigs in the AT-MSCs and UC-MSCs groups (> 83% of the pigs), indicating a superior regeneration process. The presence of inflammatory infiltration was observed in only one pig each in the HA and AT-MSC-EVs groups, indicating ongoing chronic inflammation (Table 2).

**Table 2:** Microscopic examination of the presence of thickening of the bone trabeculae (indicating bone remodeling) and inflammatory infiltration in the sections of the bone-cartilage tissue

Group	No. of pigs with detected thickening of the bone trabeculae (*n*)/*N*		No. of pigs with detected inflammatory infiltration (*n*)/*N*	
	*n*/*N*	%	*n*/*N*	%
Control	3/6	50.0	0/6	0.0
HA	4/6	66.7	1/6	16.7
AT-MSCs	6/7	85.7	0/7	0.0
UC-MSCs	5/6	83.3	0/6	0.0
AT-MSC-EVs	2/5	40.0	1/5	20.0
UC-MSC-EVs	2/4	50.0	0/4	0.0

After H&E staining, tissue sections (collected from the leg of the injured bone and cartilage) were analyzed for the presence of thickening of the bone trabeculae and inflammatory infiltration by microscopic observation. The data are presented as the number of pigs with detected thickening of the bone trabeculae/inflammatory infiltration (n) per the number of pigs in the group (N)

**Fig. 6:**
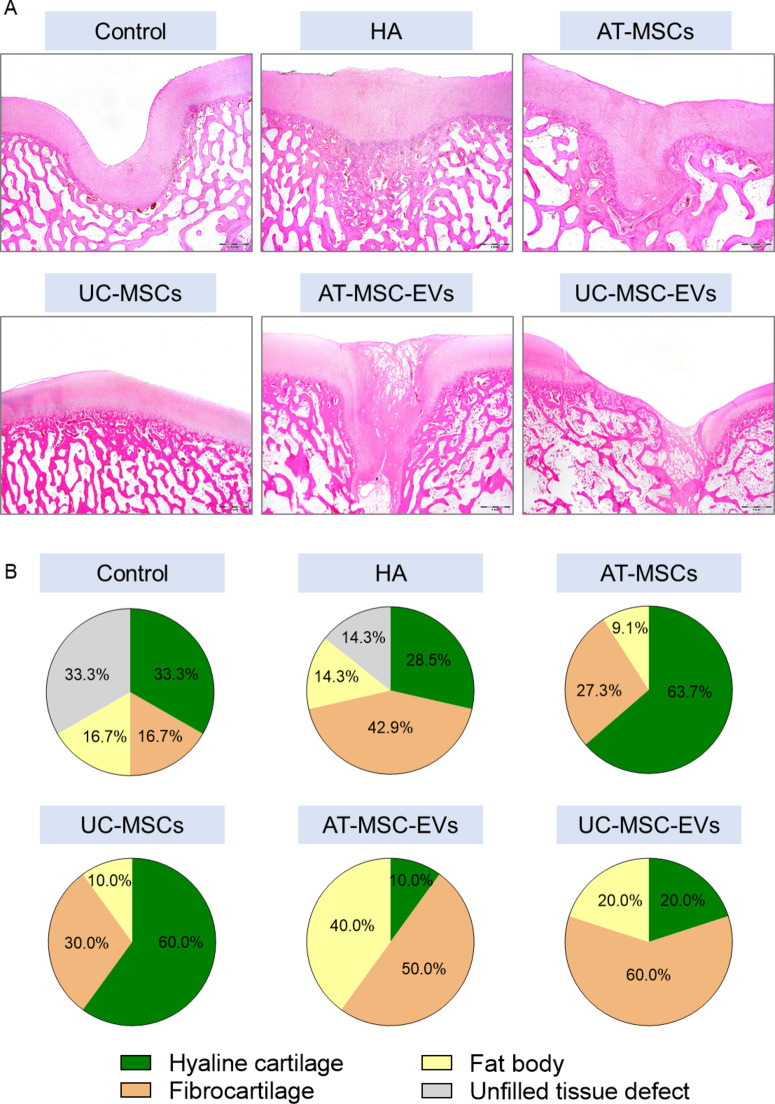
H&E staining of tissue sections of femoral condyle fragments. Transgenic pigs underwent surgical procedures to induce mechanical cartilage-bone injuries of the femoral bone in the knee and then either left to self-heal (Control) or followed by injection of HA (carrier solution mixed with HA), or with AT-MSCs, UC-MSCs, AT-MSC-EVs or UC-MSC-EVs resuspended in carrier solution and further mixed with HA. After 6 m of follow-up, cartilage-bone samples from the femoral condyles were collected, fixed with buffered 10% formalin pH 7.4 and stained with H&E. **A** Representative images of tissue sections from pigs in the experimental groups. **B** Pie charts showing percentage differences in the types of newly formed tissues filling the cartilage-bone defect between the Control and experimental groups. *N* = 4–7 pigs/group (as indicated in Table 2)

Importantly, after a 6-m follow-up period, in 63.7 and 60.0% of pigs in the AT-MSCs and UC-MSCs groups, respectively, we observed almost-fully-healed defects with newly formed hyaline cartilage, as shown by histological analysis (Fig. 6A, B). We also detected the formation of fibrocartilage in 27.3 and 30.0% of pigs in the AT-MSCs and UC-MSCs groups, respectively. In pigs in the Control group, the defects were filled with hyaline cartilage (33.3% of pigs), fibrocartilage (16.7% of pigs), or fat bodies (16.7% of pigs) or were unfilled (33.3% of pigs), whereas in pigs in the HA group, the defects were filled with hyaline cartilage (28.5% of pigs), fibrocartilage (42.9% of pigs), or fat bodies (14.3% of pigs) or were unfilled (14.3% of pigs). The percentage of unfilled defects was visibly greater in pigs in the Control group. Fibrocartilage formation was promoted in 50.0 and 60.0% of pigs in the AT-MSC-EVs and UC-MSC-EVs groups, respectively (Fig. 6B). Thus, treatment of cartilage-bone defects with cell-based products seems to be crucial for the induction of hyaline cartilage formation.

In addition to examining the pro-regenerative capacity of products containing AT-MSCs, UC-MSCs, AT-MSC-EVs, and UC-MSC-EVs, and of the control product (carrier solution mixed with high-molecular-weight HA), the safety of these products was evaluated by macroscopic and microscopic observations. No neoplasms or other potential side effects related to the administered products were observed in any pigs in the AT-MSCs, UC-MSCs, and HA groups, confirming the safety of both MSC populations and the control product (HA). No neoplasms were detected in pigs in the AT-MSC-EVs and UC-MSC-EVs groups. However, in two pigs in each of these groups, a supposed overgrowth of cartilage was observed, indicating that MSC-EVs represent biologically active particles but that the intraarticular site of administration or the dose used in this study may not be optimal for the induction of proper tissue regeneration. A summary of the safety profiles of the experimental/control products is presented in Table 3.

In summary, data from experiments in pigs indicate the safety and pro-regenerative activity on cartilage tissue of AT-MSCs and UC-MSCs, favoring not only the healing process but also the formation of hyaline cartilage, which represents the normal type of cartilage tissue on a joint bone surface. However, superior pro-regenerative capacity was observed for AT-MSCs. MSC-EVs may also be used to treat tissue injury, but their application requires additional extensive research, including determining the effective dose, route of administration, and type of carrier in the selected intended use.

**Table 3:** Macro- and microscopic examination of the presence of neoplasms and other potential side effects related to the injected products in bone-cartilage tissue sections

Method of assessment	Macro- and microscopic observations		
	Group	No. of neoplasm events/N	No. of other potential side effects related to the injected product/N
Micro-CT	Control	0/4	0/4
	HA	0/4	0/4
	AT-MSCs	0/6	0/6
	UC-MSCs	0/6	0/6
	AT-MSC-EVs	0/5	2^*^/5
	UC-MSC-EVs	0/4	2^*^/4
Histology	Control	0/6	0/6
	HA	0/6	0/6
	AT-MSCs	0/7	0/7
	UC-MSCs	0/6	0/6
	AT-MSC-EVs	0/5	0/5
	UC-MSC-EVs	0/4	0/4

Tissue sections (collected from the leg with induced bone-cartilage injury) were subjected to micro-CT scanning or H&E staining, and subsequently, during analysis of the obtained data, the presence of neoplasms and other potential side effects related to the injected product were examined by direct macro- and microscopic observations. The data are presented as the number of pigs with detected neoplasms/other potential side effects related to the injected product per the number of pigs in the analyzed group (N)

*N*– number of examined pigs (samples) per experimental group. Due to technical reasons, the number of pigs/samples per experimental group differed between the assessment methods used

(*) Overgrowth of cartilage was observed on the side of the sample

## Discussion

The purpose of the present study was to preclinically evaluate the regenerative potential of AT- and UC-derived MSCs and their derivatives (such as MSC-EVs) for the treatment of cartilage-bone injury compared with the commonly used orthopedic hyaluronic acid (HA) in a transgenic pig model to identify safe and effective products for further clinical trials in humans.

In the first stage of the study, we established optimized protocols (meeting GMP requirements) for the isolation and culture of MSCs derived from adult tissues such as AT and tissues associated with neonatal birth, such as UC, which are the main sources of MSCs [22]. The identities of both AT-derived cells and UC-derived cells isolated as MSCs were successfully confirmed according to ISCT recommendations [13]. Notably, AT collection through liposuction is relatively noninvasive for patients, and AT represents often medical waste from various surgical/esthetic medicine procedures. In addition, UC also represents post-delivery medical waste. In adult recipients, AT-derived MSCs can be used in autologous or allogenic clinical applications, whereas UC-MSCs are used mainly in allogenic transplants. It should always be considered that, depending on the desired use of MSCs (or even MSC-EVs) in distinct tissue injury treatments, protocols for the isolation and culture of MSCs need to be set up and specifically optimized to obtain cells with the most optimal biological properties and therapeutic potential [23]. Importantly, the capacity of the AT-MSCs and UC-MSCs to differentiate into chondroblasts/chondrocytes as well as osteoblasts/osteocytes, along with their potential to secrete MSC-EVs, as major mechanisms of MSC action in injured tissues [12], confirmed the rationale of this study aiming bone/ cartilage repair in OA.

MSC-EVs were isolated by differential centrifugation using a protocol optimized in our laboratory in accordance with the recommendations of the ISEV, which allowed us to isolate a heterogenous population of small and medium/large EVs from both populations of MSCs [20, 24, 25]. Subsequently, we found that isolates of both AT-MSC-EVs and UC-MSC-EVs represented heterogeneous populations of particles with sizes ranging from approximately 80–430 nm, carrying parental cell-specific antigens (i.e., CD73, CD90, and CD105) and tetraspanins (i.e., CD63 and CD81) and not possessing hematopoietic cell markers (i.e., CD34 and CD45) on their surface, confirming their MSC-EV identity. The particle size distributions and antigenic profiles of both MSC-EVs were similar to the ones we observed in our other studies [25, 26].

The safety and biological potential of cell-based (containing AT-MSCs or UC-MSCs as active substances) or cell-free (containing AT-MSC-EVs or UC-MSC-EVs as active substances) products were evaluated by intraarticular injection in a large preclinical pig model of cartilage-bone injury in vivo. In the present study, genetically modified pigs were used to eliminate the risk of acute (or chronic) cellular rejection. These animals can also be used as organ donors for xenotransplantation [27]. Furthermore, in a pig model of cartilage-bone defect, the proportions of cartilage-bone defects are closer to the dimensions of human defects than are those in other animal models in vivo [28].

After 6-m of follow-up, we observed almost-fully-healed cartilage-bone defects mainly in two groups of pigs: the AT-MSCs and UC-MSCs groups, when compared to pigs in the other groups. Importantly, cartilage and bone defects were almost never observed in pigs in the AT-MSCs group, indicating superior tissue regeneration. The volumes of cartilage and bone defects were also low in pigs in the UC-MSCs groups, suggesting effective tissue healing. Pigs in the HA group presented a limited regeneration capacity; i.e., tissue defects were still detected on the surface of the joints. Moreover, the volume of cartilage defects in HA group was comparable to the control untreated group, whereas the volume of bone defects was smaller than that in pigs in the Control group, suggesting that the treatment only with HA - without biologically active MSCs or EVs, was not sufficient to repair the defects and regenerate damaged tissues. Furthermore, we observed a lower degree of cartilage-bone tissue regeneration in the pigs in the AT-MSC-EVs and UC-MSC-EVs groups.

Importantly, in pigs in the AT-MSCs and UC-MSCs groups, approximately 60.0% of the observed almost-fully-healed tissue defects were filled with newly formed hyaline cartilage, while only approximately 30.0% were filled with fibrocartilage. On the other hand, fibrocartilage formation was promoted in the AT-MSC-EVs and UC-MSC-EVs groups (in 50.0 and 60.0% of pigs, respectively). In pigs in the untreated Control group, the defects were unhealed in about 30% of animals or filled with hyaline cartilage (30%), fibrocartilage (17%) or fat bodies, whereas in pigs in the HA group, only about 15% of pigs was unhealed or the defects were filled with hyaline cartilage (30% of pigs), fibrocartilage (40%) or fat bodies, suggesting that HA administration (without biologically active MSCs or MSC-EVs) may promote the formation of fibrocartilage. The greatest cartilage thickness above the bone defects was observed in pigs in the AT-MSCs, UC-MSCs, AT-MSC-EVs and UC-MSC-EVs groups, indicating that the treatment of cartilage-bone injury with MSC-based products (especially cell-based medicinal products) seems to be crucial for induction of tissue repair.

Thus, data from experiments in pigs indicate the regenerative activity of both populations of MSCs in cartilage tissue, which favors not only the healing process but also the formation of hyaline cartilage, which represents a normal type of cartilage tissue on a joint bone surface. No currently available repair therapy (such as autologous chondrocyte implantation, microfracture, drilling, mosaicplasty, and allograft transplantation) recreates native hyaline cartilage and provides long-term restoration due to mainly the formation of fibrocartilage and/or poor matrix properties [19, 29, 30]. This highlights the need to develop a new therapy that promotes the formation of hyaline cartilage at the site of tissue damage, which was undertaken in this study. Attempts to create such solutions using MSCs have been made by several research groups [31–33]. In patients with knee OA, bone marrow (BM)-derived MSCs embedded in collagen gel were transplanted into the articular cartilage defect in the medial femoral condyle and covered with autologous periosteum, improving the arthroscopic and histological grading scores compared with those of the cell-free control group [31]. Furthermore, in a canine model of mechanically induced cartilage defects, BM-MSCs in combination with HA injected into the knee joint cavity, regenerated more cartilage-like tissue than did HA alone or saline (significant improvement in cartilage defect regeneration was observed after BM-MSC treatment). The cartilage-bone defects were filled with neocartilage (of an unspecified kind) along with the formation of chondrocytes, whereas in HA-treated animals, some tissue similar to cartilage and fibrous tissue was observed. However, in untreated animals, the formation of neocartilage was rarely observed at the defect site [32]. Interestingly, Lin et al. compared the pro-regenerative potential of human AT-MSCs and pig UC-MSCs in a porcine articular cartilage defect model and revealed that the human AT-MSCs were more effective in repairing cartilage than pig UC-MSCs, suggesting that MSC-based treatments (especially with cells derived from AT) hold promise as options for cartilage repair, which could aid in the treatment of OA [33]. This is consistent with results from the current study where MSCs isolated from AT and UC were used.

Although AT-MSCs and UC-MSCs have been shown to exhibit a similar chondrogenic potential, UC-MSCs showed a lower osteogenic differentiation capacity when compared to AT-MSCs in vitro [34, 35]. In our study, after 6-m of follow up, we still observed slightly larger remnant bone defects in UC-MSC group when compared to AT-MSC group, which may be the result of lower osteogenic potential of UC-MSCs. It should be taken into account that MSCs are alive cells, and their mechanisms of action may be based on their direct differentiation into bone and cartilage cells, along with their paracrine activity [12]. In a rat model of OA, Ju et al. have demonstrated that both AT-MSCs and UC-MSCs inhibited OA progression after the intraarticular cell injection as well as significantly protected chondrocytes against apoptosis [35]. In other in vitro and in vivo studies, EVs released by MSC and representing non-viable objects, have been shown to increase chondrocyte proliferation and migration, inhibit apoptosis and stimulate chondrogenic differentiation of endogenous progenitor/stem cells, suggesting their paracrine mode of action [36–39]. Furthermore, EVs have been reported to increase the expression of hyaline cartilage components such as aggrecan and type II collagen at both the gene and protein levels, while reducing the expression and secretion of matrix-degrading enzymes, including matrix metalloproteinases (MMPs) and ADAMTS5 in chondrocytes in vitro and in knee tissues in vivo [38, 40–42]. However, the precise mechanism of EV action during cartilage and bone regeneration is still not well investigated. Moreover, the numerous parameters of EV-based medicinal products still need to be optimized (including e.g. source of EVs, their isolation method, dosage) prior clinical translation of EVs to achieve high outcome in patients [37].

The cartilage degeneration process in OA may be evaluated and graded based on a modified G0–G3 Outerbridge classification [43], where a higher grade corresponds to more extensive cartilage surface disruption (i.e., G0 represents pristine cartilage with no visible breaks in the surface, whereas G3 is characterized by marked softness to touch and deep and relatively wide disruption of the cartilage) [43]. It has been experimentally shown that the thickness of the SBP is significantly elevated in patients with G2 and G3 of OA [44]. In our study, we observed a significant increase in the thickness of the SBP only in pigs in the Control and HA groups (compared with pigs in the MSC- and EV- treated groups), which may suggest further degenerative changes in cartilage and bone tissue in untreated or HA- treated animals, along with the cessation of OA progression in MSC- and MSC-EV-treated pigs. Furthermore, the BMD of the SBP and trabecular bone was slightly elevated in pigs in the Control and HA groups compared with those in the other groups, which corresponds to adverse progression in OA. Interestingly, the elevated BMD values of the SBP and the trabecular bone were also observed in healthy unoperated joints (and were similar to those for the operated joints of pigs in the Control and HA groups), which may be caused by the relieve of the injured limb and overweighting the healthy leg by the injured animal. Moreover, in pigs in these groups, the other parameter - the ratio of bone volume to total sample volume (BV/TV), was significantly higher than that in the unoperated joints and in pigs in the MSC and MSC-EV groups, indicating also ongoing degenerative changes in cartilage-bone tissues. It has been shown that patients with OA exhibit a 15% higher bone density and a 30% higher bone volume than healthy controls [3], which is consistent with our observations in the pig model. In contrast, in pigs in the AT-MSCs, UC-MSCs, AT-MSC-EVs and UC-MSC-EVs groups, the thickness of the SBP fell within the normal range for these animals. The current study confirmed the beneficial effect of MSCs and MSC-EVs on SBP and trabecular bone indicating their positive impact on regeneration process. Moreover, in our study, the percentage of pigs that showed thickening of the bone trabeculae was the highest among the animals in the AT-MSCs and UC-MSCs groups, indicating a superior regeneration process of subchondral bone in these groups of animals. It should be taken into consideration that subchondral bone may impact on cartilage regeneration: the subchondral bone not only nourishes the cartilage, but also stabilizes it and supplies endogenous MSCs and the growth factors [45]. Moreover, it has been reported that changes in the metabolism of the subchondral bone are an integral part of the OA development and progression. Thus, there is a strong rationale for developing therapeutic approaches that behind articular cartilage may also target subchondral bone [21, 46].

Although we did not observe a high degree of regeneration of cartilage-bone defects after intraarticular injection of products containing both types of MSC-EVs, the pro-regenerative potential of human MSC-derived exosomes (the fraction of smaller MSC-EVs) in the treatment of osteochondral defects was presented by Wong et al., who reported that tissue defects were filled with newly repaired tissues composed mainly of hyaline cartilage along with improvement in the mechanical properties of regenerated cartilage [47]. This finding indicates that the MSC-EVs tested in the present study may be used to treat tissue injury in the future, but their application requires additional extensive research and optimization, including determining the effective dose, route of administration and type of carrier in the selected intended treatment, which steps are required by the European Medicines Agency when designing advanced therapy medical products (ATMPs) (https://www.ema.europa.eu).

With respect to safety, we did not observe the formation of neoplasms or other side effects related to the injected products in pigs in the AT-MSCs and UC-MSCs groups. The meta-analysis conducted by Wang et al. covered 62 randomized clinical trials using MSCs in various indications and clearly confirmed that MSC administration was safe in patients when compared with other placebo modalities [48]. MSC-EVs are also known for their confirmed safety profile [49], which was also observed in our study. However, in two pigs (each in the AT-MSC-EVs and UC-MSC-EVs groups), a supposed overgrowth of cartilage was observed, indicating that MSC-EVs represent biologically active particles stimulating cartilage growth, but that the intraarticular site of administration or the dose used in this study may not be optimal for the induction of proper tissue formation and requires further investigations.

In summary, our in vivo data indicate the safety and pro-regenerative activity on cartilage tissue of AT-MSCs and UC-MSCs, favoring not only the healing process but also the formation of hyaline cartilage, which represents the normal type of cartilage tissue on the joint bone surface. A superior pro-regenerative capacity was observed for AT-MSCs, which were subsequently chosen as an active substance for an ATMP product for clinical trials using patients with OA. The use of MSC-EVs for the treatment of cartilage-bone defects needs further evaluation. MSCs and MSC-EVs may also exert beneficial effects on the SBP and trabecular bone, which should also be considered as a target for effective OA therapy. To conclude, the treatment of cartilage-bone defects with products containing MSCs as an active substance favors the healing process of injured tissues along with the formation of hyaline cartilage, which represents the normal type of cartilage tissue on a joint bone surface. MSCs represent promising candidates for effective and safe biologically active substances that may be used in the development of ATMPs for use in humans.

## Electronic Supplementary Material

Below is the link to the electronic supplementary material.

Supplementary Material 1

## Data Availability

The underlying data for this article can be found in the article and its Supplementary file.

## Declarations

Human umbilical cords were provided by Polish Stem Cell Bank LLC, with permission from the Polish Ministry of Health (MZ-PZ-TSZ-025-15906-36/AB/14), obtained based on the approval of the appropriate Bioethics Committee. Written informed consent was obtained for participation in the study, for the use of biological material and for the publication of results from umbilical cord donors. The Material Transfer Agreement (free of charge) was signed by the Polish Stem Cell Bank and Jagiellonian University.

### Use of AI

The authors declare that they have not used generative AI in the preparation of this manuscript.

## Abbreviations

ATMPs: Advanced therapy medicinal products
AT-MSCs: Adipose tissue-derived mesenchymal stem/stromal cells
AT-MSC-EVs: Extracellular vesicles from adipose tissue-derived mesenchymal stem/stromal cells
BMD: Bone mineral density
EVs: Extracellular vesicles
HA: Hyaluronic acid
MSCs: Mesenchymal stem/stromal cells
OA: Osteoarthritis
SBP: Subchondral bone plate
UC-MSCs: Umbilical cord-derived mesenchymal stem/stromal cells
UC-MSC-EVs: Extracellular vesicles from umbilical cord-derived mesenchymal stem/stromal cells
