# Supplementary material for: Evaluation of the Safety and Regenerative Potential of Human Mesenchymal Stem Cells and Their Extracellular Vesicles in a Transgenic Pig Model of Cartilage-Bone Injury In Vivo – Preclinical Study
Source: Stem Cell Rev Rep. 2025 May 17;21(4):1075–95. doi: 10.1007/s12015-025-10853-4 (PMC12102096; doi:10.1007/s12015-025-10853-4)
Supplement: Supplementary file 1 — Supplementary Material 1 [file 12015_2025_10853_MOESM1_ESM.pdf]

Stem Cell Reviews and Reports

Supplementary File 1

Evaluation of the safety and regenerative potential of human mesenchymal stem cells and their extracellular vesicles in a transgenic pig model of cartilage-bone injury *in vivo* – preclinical study

Anna Łabędź-Masłowska[*,1], Jarosław Wieczorek[*,2], Maciej Mierzwiński[3], Małgorzata Sekuła-Stryjewska[1,4], Sylwia Noga[1,4], Jolanta Rajca[3,5], Piotr Duda[6], Katarzyna Milian-Ciesielska[7], Elżbieta Karnas[1], Katarzyna Kmiotek-Caller[1], Agnieszka Szkaradek[1], Zbigniew Madeja[1], Krzysztof Ficek[3], Jacek Jura[#,8] Ewa Zuba-Surma [#,1]

[1]Department of Cell Biology, Faculty of Biochemistry, Biophysics and Biotechnology, Jagiellonian University, Krakow, Poland.

[2]University Center of Veterinary Medicine UJ-UR, University of Agriculture in Krakow, Krakow, Poland.

[3]Department of Science, Innovation and Development, Galen-Orthopaedics, Bierun, Poland.

[4]Malopolska Centre of Biotechnology, Jagiellonian University, Krakow, Poland.

[5]Spin-Lab Centre for Microscopic Research on Matter, University of Silesia in Katowice, Katowice, Poland.

[6]Institute of Biomedical Engineering, Faculty of Science and Technology, University of Silesia in Katowice, Katowice, Poland.

[7]Department of Pathomorphology, Jagiellonian University Medical College, University Hospital, Krakow, Poland.

[8]Department of Reproductive Biotechnology and Cryoconservation, National Research Institute of Animal Production, Balice, Poland.

[*]First coauthors.

[#]Corresponding coauthors.

Correspondence: Ewa Zuba-Surma e-mail: ewa.zuba-surma@uj.edu.pl

Jacek Jura e-mail: jacek.jura@iz.edu.pl

Figure S1

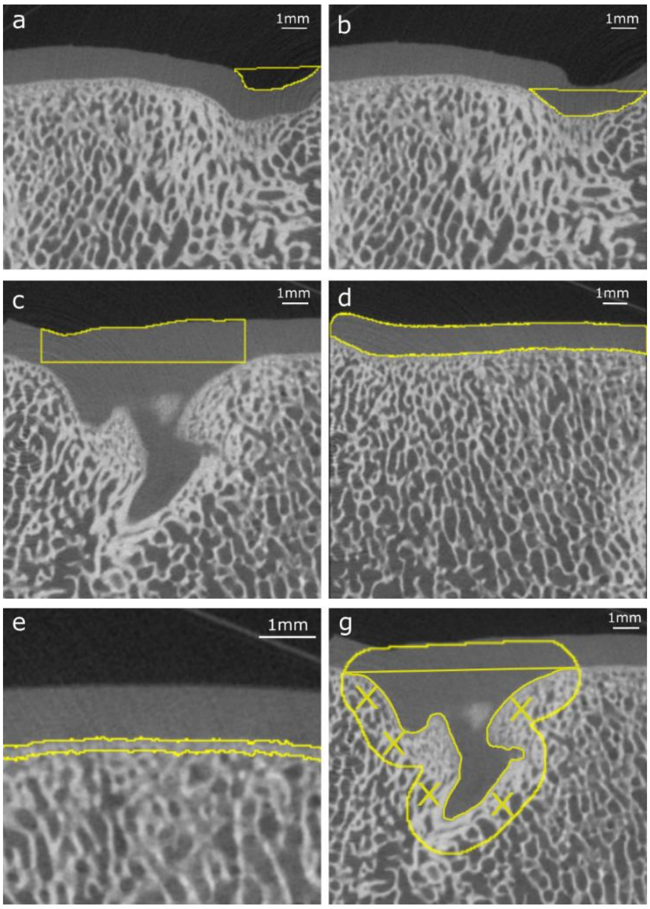

Fig. S1. ImageJ analysis of cartilage-bone samples. The volume of interest (VOI) marked for: a) cartilage defect, b) bone defect, c) cartilage thickness above bone defect, d) cartilage thickness above healthy bone (in unaffected region), e) subchondral bone plate thickness, f) the trabecular bone around the defect (X-marked VOI).

Figure S2

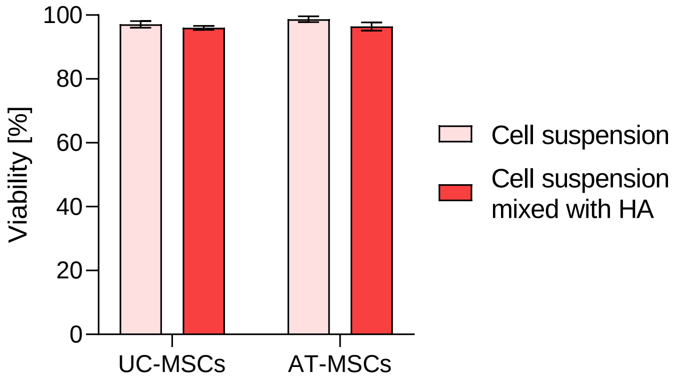

Fig. S2. Viability of AT-MSCs and UC-MSCs before and after mixing with high molecular weight hyaluronic acid (HA) by trypan blue dye exclusion method.

Table S1. Summary data for analysis of volume of cartilage defect. *ns- non-significant.*

Unit no.	Volume of cartilage defect [mm^3]					
	Group of animals					
	Control	HA	AT-MSCs	UC-MSCs	AT-MSC-EVs	UC-MSC-EVs
1	8.09	0.00	1.17	14.71	24.18	20.32
2	9.02	0.00	0.00	0.00	3.67	2.15
3	7.83	26.89	0.18	0.00	0.00	0.00
4	16.11	11.26	0.00	0.00	34.76	0.00
5			0.00	0.00	0.00	
6			0.00	0.00		
N	4	4	6	6	5	4
**Mean**	**10.26**	**9.54**	**0.23**	**2.45**	**12.52**	**5.62**
SD	3.93	12.73	0.47	6.01	15.99	9.85
p vs. Control	-	*ns*	*<0.01*	*<0.05*	*ns*	*ns*
p vs. HA	*ns*	-	*ns*	*ns*	*ns*	*ns*

Table S2. Summary data for analysis of volume of bone defect. *ns- non-significant.*

Unit no.	Volume of bone defect [mm^3]					
	Group of animals					
	Control	HA	AT-MSCs	UC-MSCs	AT-MSC-EVs	UC-MSC-EVs
1	49.60	13.76	4.05	20.01	74.11	22.35
2	84.06	0.50	0.00	0.65	77.00	85.08
3	19.67	70.14	0.83	35.38	0.00	0.00
4	22.36	10.46	0.00	0.00	6.70	0.00
5			0.00	0.00	52.05	
6			0.00	4.56		
N	4	4	6	6	5	4
**Mean**	**43.92**	**23.72**	**0.81**	**10.10**	**41.97**	**26.86**
SD	29.98	31.46	1.62	14.57	36.63	40.22
p vs. Control	-	*ns*	*<0.01*	*ns*	*ns*	*ns*
p vs. HA	*ns*	-	*<0.05*	*ns*	*ns*	*ns*

Table S3. Summary data for analysis of cartilage thickness above healthy bone. *ns- non-significant.*

Unit no.	Cartilage thickness above healthy bone [mm]					
	Group of animals					
	Control	HA	AT-MSCs	UC-MSCs	AT-MSC-EVs	UC-MSC-EVs
1	1.12	1.26	1.22	1.35	0.95	1.64
2	1.14	1.05	1.43	1.14	1.45	1.29
3	1.08	1.17	1.04	1.36	1.00	1.30
4	1.05	1.10	0.83	1.13	1.41	1.19
5			1.47	0.70	1.17	
6			0.94	1.05		
N	4	4	6	6	5	4
**Mean**	**1.10**	**1.15**	**1.16**	**1.12**	**1.20**	**1.36**
SD	0.04	0.09	0.26	0.24	0.23	0.20
p vs. Control	-	*ns*	*ns*	*ns*	*ns*	*ns*
p vs. HA	*ns*	-	*ns*	*ns*	*ns*	*ns*

Table S4. Summary data for analysis of cartilage thickness above bone defect. *ns- non-significant.*

Unit no.	Cartilage thickness above bone defect [mm]					
	Group of animals					
	Control	HA	AT-MSCs	UC-MSCs	AT-MSC-EVs	UC-MSC-EVs
1	0.00	1.26	1.08	0.00	0.00	0.00
2	0.90	0.88	1.43	1.14	0.90	0.91
3	0.00	0.00	0.46	1.36	1.00	1.30
4	0.46	0.00	0.83	1.13	1.26	1.19
5			1.47	0.70	1.36	
6			0.94	1.05		
N	4	4	6	6	5	4
**Mean**	**0.34**	**0.54**	**1.04**	**0.90**	**0.90**	**0.85**
SD	0.43	0.64	0.38	0.49	0.54	0.59
p vs. Control	-	*ns*	*<0.01*	*<0.01*	*<0.01*	*<0.01*
p vs. HA	*ns*	-	*ns*	*ns*	*ns*	*ns*

Table S5. Summary data for analysis of subchondral bone plate (SBP) – bone mineral density (BMD). *ns- non-significant.*

Unit no.	Subchondral bone plate - BMD [g Hap/cm^3]						
	Group of animals						
	Healthy limb	Control	HA	AT-MSCs	UC-MSCs	AT-MSC-EVs	UC-MSC-EVs
1	0.65	0.67	0.68	0.6	0.52	0.69	0.61
2	0.76	0.72	0.64	0.62	0.6	0.63	0.65
3	0.69	0.59	0.63	0.70	0.61	0.61	0.64
4	0.69	0.69	0.63	0.60	0.6	0.64	0.68
5	0.65			0.50	0.65	0.60	
6	0.58			0.68	0.6		
N	6	4	4	6	6	5	4
**Mean**	**0.67**	**0.67**	**0.65**	**0.62**	**0.60**	**0.63**	**0.65**
SD	0.06	0.06	0.02	0.07	0.04	0.04	0.03
p vs. Healthy limb	-	*ns*	*ns*	*ns*	*ns*	*ns*	*ns*
p vs. Control	*ns*	-	*ns*	*ns*	*ns*	*ns*	*ns*
p vs. HA	*ns*	*ns*	-	*ns*	*ns*	*ns*	*ns*

Table S6. Summary data for analysis of subchondral bone plate (SBP) - thickness. *ns- non-significant.*

Unit no.	Subchondral bone plate - thickness [mm]						
	Group of animals						
	Healthy limb	Control	HA	AT-MSCs	UC-MSCs	AT-MSC-EVs	UC-MSC-EVs
1	0.19	0.36	0.36	0.22	0.18	0.23	0.20
2	0.13	0.36	0.36	0.21	0.23	0.16	0.14
3	0.19	0.29	0.31	0.25	0.20	0.18	0.19
4	0.15	0.37	0.32	0.18	0.18	0.23	0.19
5	0.14			0.24	0.11	0.19	
6	0.16			0.17	0.21		
N	6	4	4	6	6	5	4
**Mean**	**0.16**	**0.35**	**0.34**	**0.21**	**0.19**	**0.20**	**0.18**
SD	0.03	0.04	0.03	0.03	0.04	0.03	0.03
p vs. Healthy limb	-	*ns*	*ns*	*<0.05*	*ns*	*ns*	*ns*
p vs. Control	*<0.01*	-	*ns*	*<0.01*	*<0.01*	*<0.01*	*<0.05*
p vs. HA	*<0.01*	*ns*	-	*<0.01*	*<0.01*	*<0.01*	*<0.05*

Table S7. Summary data for analysis of trabecular bone – bone mineral density (BMD). *ns- non-significant.*

Unit no.	Trabecular bone - BMD [g Hap/cm^3]						
	Group of animals						
	Healthy limb	Control	HA	AT-MSCs	UC-MSCs	AT-MSC-EVs	UC-MSC-EVs
1	0.77	0.76	0.82	0.62	0.65	0.83	0.83
2	0.76	0.70	0.69	0.72	0.68	0.74	0.88
3	0.81	0.76	0.76	0.74	0.79	0.73	0.74
4	0.76	0.91	0.73	0.78	0.63	0.90	0.71
5	0.79			0.51	0.71	0.79	
6	0.70			0.75	0.60		
N	6	4	4	6	6	5	4
**Mean**	**0.76**	**0.78**	**0.75**	**0.69**	**0.68**	**0.80**	**0.79**
SD	0.04	0.09	0.05	0.10	0.07	0.07	0.08
p vs. Healthy limb	-	*ns*	*ns*	*ns*	*ns*	*ns*	*ns*
p vs. Control	*ns*	-	*ns*	*ns*	*ns*	*ns*	*ns*
p vs. HA	*ns*	*ns*	-	*ns*	*ns*	*ns*	*ns*

Table S8. Summary data for analysis of trabecular bone – bone to total volume ratio (BV/TV). *ns- non-significant.*

Unit no.	Trabecular bone – BV/TV [%]						
	Group of animals						
	Healthy limb	Control	HA	AT-MSCs	UC-MSCs	AT-MSC-EVs	UC-MSC-EVs
1	59.39	59.71	69.99	63.10	68.57	57.19	75.76
2	56.18	73.02	70.13	56.89	70.25	57.98	68.92
3	56.39	69.07	57.32	87.24	80.94	57.67	56.61
4	60.63	76.13	74.75	62.83	53.52	69.98	60.41
5	49.52			50.99	37.00	71.37	
6	54.04			59.42	70.38		
N	6	4	4	6	6	5	4
**Mean**	**56.03**	**69.48**	**68.05**	**63.41**	**63.44**	**62.84**	**65.43**
SD	3.97	7.13	7.49	12.50	15.65	7.18	8.60
p vs. Healthy limb	-	*ns*	*ns*	*ns*	*ns*	*ns*	*ns*
p vs. Control	*<0.05*	-	*ns*	*ns*	*ns*	*ns*	*ns*
p vs. HA	*<0.05*	*ns*	-	*ns*	*ns*	*ns*	*ns*
